# A qualitative study of community perspectives surrounding cleaning practices in the context of Zika prevention in El Salvador: implications for community-based *Aedes aegypti* control

**DOI:** 10.1186/s12889-020-09370-5

**Published:** 2020-09-11

**Authors:** Elli Leontsini, Sean Maloney, Margarita Ramírez, Eric Rodriguez, Tilly Gurman, Anne Ballard Sara, Gabrielle C. Hunter

**Affiliations:** 1 Department of International Health, Johns Hopkins Bloomberg School of Public Health, 615 N. Wolfe St, Baltimore, MD 21205 USA; 2 Johns Hopkins Center for Communication Programs, Bloomberg School of Public Health, 111 Market Place, Suite 310, Baltimore, MD 21202 USA; 3 Bulevar Acatán 31-25 zona 16, Villas de San Isidro, Torre I, Apto 201, 01016 Guatemala City, Guatemala

**Keywords:** Zika, Dengue, Chikungunya, Water storage containers, Behavioral determinants, Mosquito ovicidal technique, *Untadita*, Community control of *Aedes aegypti*, Arbovirus prevention, El Salvador

## Abstract

**Background:**

In El Salvador, *Aedes aegypti* mosquitoes transmitting Zika and other arboviruses use water storage containers as important oviposition sites. Promotion of water storage container cleaning is a key element of prevention programs. We explored community perceptions surrounding cleaning practices among pregnant women, male partners of pregnant women, and women likely to become pregnant.

**Methods:**

Researchers conducted 11 focus groups and 12 in-depth interviews which included individual elicitations of Zika prevention measures practiced in the community. Focus group participants rated 18 images depicting Zika-related behaviors according to effectiveness and feasibility in the community context, discussed influencing determinants, voted on community intentions to perform prevention behaviors, and performed washbasin cleaning simulations. In-depth interviews with male partners of pregnant women used projective techniques with images to explore their perceptions on a subset of Zika prevention behaviors.

**Results:**

General cleaning of the home, to ensure a healthy environment, was a strong community norm. In this context, participants gave water storage container cleaning a high rating, for both its effectiveness and feasibility. Participants were convinced that they cleaned their water storage containers effectively against Zika, but their actual skills were inadequate to destroy *Aedes aegypti* eggs. A further constraint was the schedule of water availability. Even during pregnancy, male partners rarely cleaned water storage containers because water became available in homes when they were at work. Furthermore, prevailing gender norms did not foster male participation in domestic cleaning activities. Despite these factors, many men were willing to provide substantial support with cleaning when their partners were pregnant, in order to protect their family.

**Conclusions:**

Behavior change programs for the prevention of Zika and other arboviruses need to improve community members’ mosquito egg destruction skills rather than perpetuate the promotion of non-specific cleaning in and around the home as effective. Egg elimination must be clearly identified as the objective of water storage container maintenance and programs should highlight the effective techniques to achieve this goal. In addition, programs must build the skills of family members who support pregnant women to maintain the frequency of effective egg destruction in all water storage containers of the home.

## Background

In Latin America, the arboviruses Zika,^1^ dengue, and chikungunya are transmitted to people by infected *Aedes aegypti* (*Ae. aegypti*) mosquitoes. Primarily human biters, these proliferate in domestic water storage containers as well as rain-filled artificial containers [1]. Many Latin American households typically store water in containers in and around the home due to the lack of reliable water supply. A study on dengue among urban residents in Colombia found that out of 1721 households surveyed, 96.1% either reported storing water in containers or were observed to have water storage containers [2]. In El Salvador, water services are limited: 48% of the drinking water supply was rated as intermittent in a 2014 report sponsored by the World Bank [3]. A 2016 cross-sectional study in the San Salvador metropolitan area found that all 110 respondents used water storage containers in the home, despite 98.2% of households surveyed having piped water; 96.4% of all respondents indicated that continued use of water storage containers was due to inconsistent water supply [4].

To date, the primary measure to prevent arbovirus outbreaks is the reduction of adult mosquito vector densities. This reduction may be even more critical in the case of an emerging arbovirus among a fully susceptible population. There is evidence to suggest that water storage containers become persistent sources of *Ae. aegypti* adults, maintaining production of *Ae. aegypti* pupae throughout the year, because households use them year-round [5]. In an entomological study of vector breeding in urban settings of five Latin American countries, large water storage containers in highly dengue-endemic areas of Colombia and Ecuador were found to produce most of the *Ae. aegypti* mosquitoes, and their infrequent cleaning was identified as a key determinant of mosquito propagation [6]. For these reasons, water storage containers are critical sites for mosquito source reduction interventions.

To be effective as a mosquito control measure, cleaning must eliminate the mosquito eggs and larvae, and be feasible for those doing the cleaning. Unlike other mosquitoes such as *Culex quinquefasciatus* that lay eggs on the water surface, *Ae. aegypti* lay eggs on the interior walls of water-filled storage containers—just above the water line [7]. It therefore follows that general house cleaning, and elimination of water puddles will not control this mosquito. Cleaning of water storage containers may not eliminate all larvae and the cleaning techniques commonly used may lack the specificity needed to effectively remove Ae. aegypti eggs [8]. This because a householder’s objective may be to eliminate visible dirt such as grime or algae rather than to target and destroy *Ae. aegypti* eggs on the walls of those containers. As a result, householders may become frustrated at the persistent presence of mosquito larvae even after cleaning the container and come to doubt the effectiveness of the cleaning practice.

To meet these challenges, a study conducted in El Progreso, Honduras by Sherman et al. [8] developed the *untadita* (the little dab), an ovicidal procedure based on existing practices and locally available products. It involved dabbing a mixture of undiluted chlorine bleach and detergent at a ratio of 5:1 directly onto the walls of emptied water storage containers followed by a 10-min waiting time for the ovicidal effect to occur. The method should be performed weekly [8], to break the *Ae. aegypti* life cycle, which requires seven to 14 days to emerge as an adult after an egg hatches [7]. An evaluation of the *untadita* in a subsequent controlled trial in Honduras was shown to markedly prevent the development of adult *Ae. aegypti* mosquitoes [9]. After inception of the *untadita* in Honduras, other variations of the method have been developed. In the Dominican Republic, e.g., a simplified approach removed the detergent to reduce the number of rinses required, and used bleach only, while increasing the wait time to 15 min [10, 11]. It is important to note that bleach should be dabbed undiluted. Diluting bleach with water, pouring bleach into the water at the bottom of a container, or pouring bleach drops directly into the water to purify it, delivers the bleach at too low a concentration to destroy eggs or kill mosquito larvae [8].

El Salvador has a long history of dengue epidemics with the reported number of cases among the highest in the Americas. Between 1980 and 2007, 132,881,000 dengue fever cases and 2,073,000 dengue hemorrhagic fever cases were reported [12]. In 2002, as part of the response to the repeated dengue outbreaks, the Ministry of Public Health and Social Assistance of El Salvador, and the United States Agency for International Development (USAID)-funded CHANGE project^2^ had recommended that the *untadita* become an integral part of El Salvador’s national dengue prevention strategy [13]. Based on locally conducted formative research [14], any one of three modalities was recommended, according to the situation in each household: a) dabbing walls with undiluted detergent and bleach followed by scrubbing of the completely emptied water container; b) dabbing bleach alone on walls of the completely emptied water container; and c) dabbing bleach alone just above the water level, without emptying the container, as a temporary measure to use during periods of extreme water scarcity. All three modalities required waiting 15 min after dabbing to give the bleach enough time for the ovicidal effect to occur. Weekly application of any of the three modalities was recommended to break the *Ae. aegypti* life cycle rather than wait longer for a completely emptied container [13]. Since 2002, either the *untadita* or the washing of containers with bleach had been included in the country’s various dengue prevention strategy documents [15, 16]. Since 2015, the approach has also been promoted for chikungunya and Zika prevention efforts [17, 18].

When Zika transmission emerged in South and Central America in 2015–2016, national governments and international agencies undertook immediate action to halt its transmission. Efforts primarily involved communication activities through mass media and community mobilization to promote uptake of preventive behaviors by individuals, families, and communities. A suite of preventive behaviors with a high potential to prevent Zika and negative pregnancy outcomes includes behaviors for preventing mosquito bites, sexual transmission of Zika, and reducing *Ae. aegypti* larval habitats around the home and community [19].

In 2015–16, El Salvador experienced multiple cases of Zika, with 11,413 suspected cases recorded through the end of 2016 [20]. Fifty-one of these cases were confirmed, including four with Zika-associated congenital syndrome. Another 376 suspected cases were reported through January 4, 2018 [21]. Due to the limitations in confirmation testing and the mild symptoms of the infection, the actual number of Zika infections may have been higher during this period. Those came shortly after an unprecedented chikungunya outbreak with over 135,000 suspected cases in 2014 [22] and another 64,000 suspected cases in 2015 [23]. While knowledge, attitudes, and practice surveys have been carried out in El Salvador [24–28], only one such survey provides information on potential motivators and barriers to Zika prevention, in which perceived effectiveness and community norms may have been important motivators, while difficulty of practice a barrier [28]. Qualitative information for a more in-depth understanding of motivators and barriers to Zika prevention behaviors in the country is lacking. Specifically, how people perceive and implement the protection and cleaning of water storage containers in their daily lives, including how mosquito eggs may be removed from water storage containers, in urban and rural areas of El Salvador is not well understood.

The present qualitative study aimed to provide rich formative data on perceptions of several Zika prevention behaviors, including the *untadita* technique. We sought insights into five behavioral determinants: 1) community norms of Zika prevention, 2) perspectives on relative effectiveness of different behaviors, 3) feasibility to practice the behaviors, including men’s social support of Zika prevention in the context of urban and rural El Salvador, 4) people’s skills for effective mosquito egg removal, and 5) people’s intentions to perform the recommendations in the community context. While we conducted a comprehensive qualitative exploration of multiple Zika prevention behaviors, in this paper we focus on behaviors related to cleaning water storage containers, including the *untadita*, and the factors that influence these behaviors.

## Methods

Behavioral psychology researchers reviewing theories of health behavior change have reached a consensus that positive social pressure, significant anticipated benefits outweighing the disadvantages, lack of environmental constraints, the possession of necessary skills, and a strong positive intention to perform the behavior are among the key factors influencing behavior and behavior change [29]. Identifying and addressing these determinants might maximize the effectiveness of community-based *Ae. aegypti* control activities for Zika and other arbovirus prevention. We studied the freely elicited actions that people in the community took to prevent Zika, as a proxy for positive social pressure or norms; the relative effectiveness of Zika prevention recommendations as a proxy for their anticipated benefits; their relative feasibility, as well as seasonality and men’s social support of Zika prevention recommendations as proxies for their environmental constraints; and whether people had the skills and intentions to perform the recommendations in the community context.

We employed a qualitative study design consisting of focus groups and in-depth interviews (IDIs) in an urban and rural context of varying topography in El Salvador, in case there were differences in practices or their determinants. Focus group activities included three-pile ratings, a simulation exercise, and individual voting. IDIs included further probing on the study’s themes. The participants came from two locations, Ilobasco, Cabañas dept. and Guaymango, Ahuachapán dept. Ilobasco is a larger urban municipality located 48 km northeast of San Salvador at a medium altitude of 750 m, and Guaymango is a smaller rural agricultural community located 96 km west of San Salvador at a lower altitude of 337 m near the Guatemalan border, hitherto referred to as the urban and rural site, respectively. Both of these locations had reported Zika cases according to the Ministry of Health as well as USAID’s Zika implementing partners’ conducting behavior change interventions such as community educational meetings, talks in schools and antenatal care sessions, community clean-up campaigns, and home counseling visits. Our research team selected these communities with the support of Save the Children and USAID/El Salvador.

Save the Children staff, with the support of the respective local health center, selected and invited participants through convenience sampling from three target populations: pregnant women 18 to 30 years of age, non-pregnant women aged 18 to 30 with a stable partner and zero to one child (as a proxy for women likely to become pregnant in the near future) or an expressed desire to have another child, and men over 18 years of age with a pregnant partner at the time of the study. All interviewers and facilitators had extensive training and experience in qualitative data collection, were native or bilingual Spanish speakers and were familiar with the Central American context. Focus group and IDI field guides are provided as additional files 1, 2, 3, 4.

Before beginning the focus groups and IDIs, we asked each participant individually to mention all the actions that people in their community took to prevent Zika, recording each freely elicited action on a form, and probing to ensure complete elicitations, and the researchers’ clear understanding of the actions described by the participant.

**Fig. 1:**
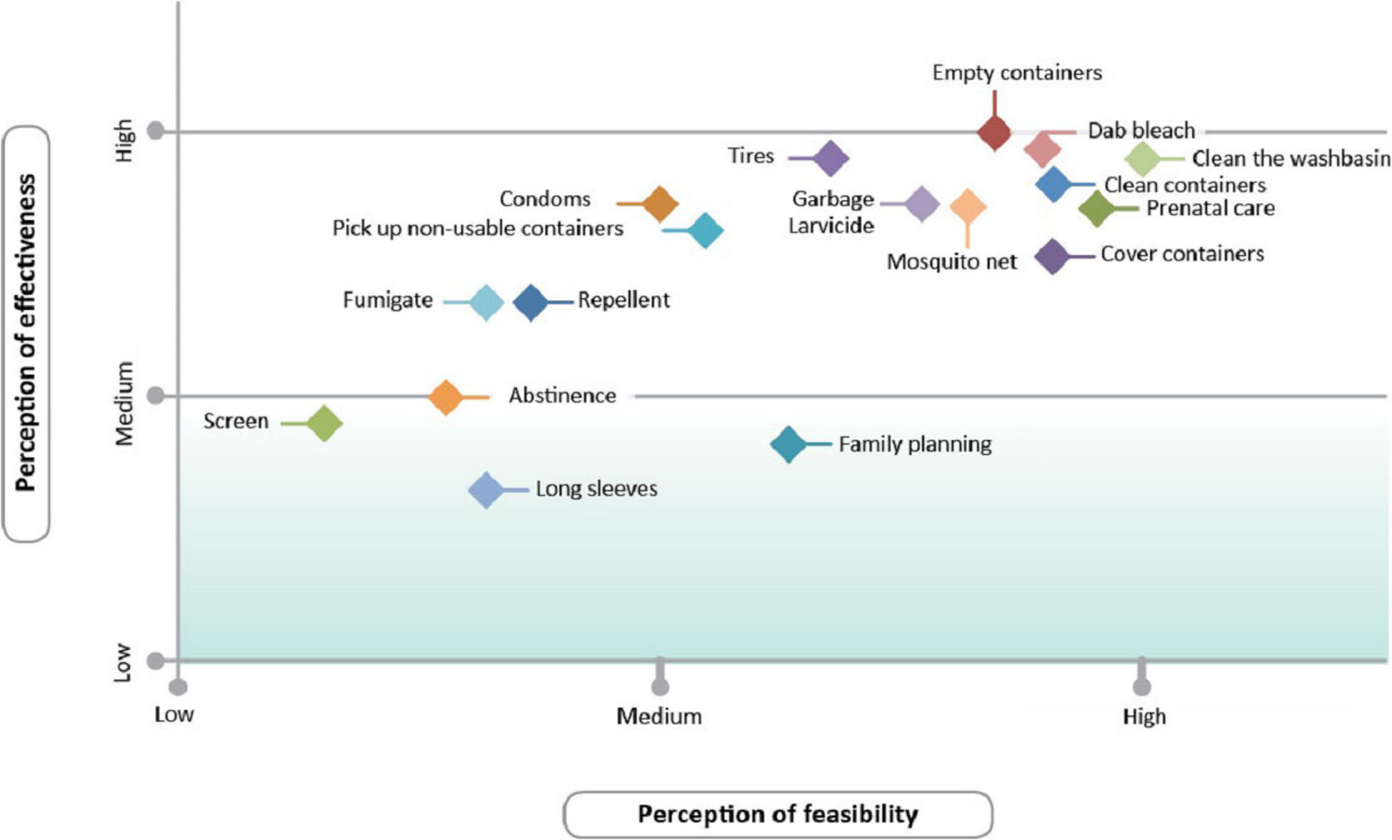
Perceived effectiveness and feasibility of 18 candidate Zika prevention behaviors rated among 11 focus groups. Vertical and horizontal coordinates represent the averaged rating for each behavior

Focus groups began with introductions and a series of group activities. First, participants engaged in a discussion of 18 images depicting Zika-related behaviors. The behaviors shown in the images represented one of three categories: personal protection, vector control, and facilitating behavioral recommendations [19], along with a few popular but ineffective vector control actions. All images are represented in Fig. 1. The moderator introduced the images not as de facto methods to prevent Zika, but as a series of actions to discuss in the focus group, in the context of Zika prevention. After discussing the behaviors, participants assigned them to three piles of low, medium, or high perceived effectiveness among their community members. Next, participants performed a second rating in which they assigned the same images to groupings of low, medium, or high feasibility to practice the behavior in the community context. Participants came to a consensus on the rating of each image and explained their reasons for disagreement, for each rating.

After the rating activities, we asked participants to individually write down or draw the materials and steps used in the community to clean the rectangular, cement washbasin, known in El Salvador as a *pila*. We invited a volunteer to simulate these steps, using a large oblong plastic container in lieu of a *pila*. Upon request, the research team provided cleaning supplies, such as water, detergent, bleach, brooms and brushes. The simulation was followed by a discussion among all participants about how their own recorded procedures complemented or deviated from those demonstrated. Before the focus group concluded, each participant voted for the three behaviors (out of the 18 discussed through the images) that they felt people in their community would be most willing to implement to prevent Zika.

We conducted IDIs with expectant fathers who had not participated in the focus groups. We utilized projective techniques with four of the Zika behavioral recommendation images used during the focus groups, to further explore the perceptions of these behaviors among men in their community. The images selected represented the main types of Zika preventive actions. Two of the images were emptying water from containers around the yard, and cleaning water storage containers (referred to in Fig. 1 as Empty containers, and Clean containers). Because women were over-represented in the focus groups, the IDIs with the men provided the opportunity to further explore gender roles and gain a deeper understanding of Zika prevention behaviors from the perspective of expectant fathers.

From April 16 to 24, 2018, we conducted 11 focus groups with 71 participants and 12 IDIs. We conducted 6 IDIs and 6 focus groups per study site, 2 focus groups with each target population, with the exception of the rural site where we conducted 1 focus group only with the men. The focus groups and IDIs were recorded on digital audio and then transcribed into text for thematic coding using qualitative analysis software (Atlas.ti). The washbasin cleaning simulations were recorded on video and then separated into individual static images for each step, using a cross-platform multimedia player (VLC Media Player). The cleaning materials and steps written down by individual participants were typed, ordered, and sorted in spreadsheet software (Microsoft Excel). The individually elicited Zika prevention actions were similarly typed and ordered, based on the frequency with which they were mentioned. The ratings chosen by each focus group on the feasibility and effectiveness of each behavior were averaged among all focus groups. The final voting was consolidated among all focus group participants. This quantification of qualitative data complemented the interpretation of the textual data on the determinants of Zika-prevention actions, however, like with any qualitative data, no population-level inferences are possible based on the quantitative measures.

We developed a codebook to guide thematic coding according to the Zika-related behavior and the determinants of its effectiveness or feasibility. The codebook is provided in additional files 5, 6. Respective quotes were sorted by study population and Zika prevention method, and various sets were printed. We held a five-day data analysis workshop from June 11 to 15, 2018, to analyze the printed quotes as a team, including team members directly involved in the data collection as well as representatives of two partners working on the Zika response in El Salvador: the United Nations Children’s Fund (UNICEF) and Medical Care Development International (MCDI). Workshop members worked in pairs, reading all the coded text and documenting key findings regarding determinants of the prevention behaviors and selecting key quotes for each behavior and study population. Pairs then shared their analyses with the larger group for discussion and identified trends across the data. After the workshop, we used the written and photographic data generated by the washbasin cleaning simulations, and ordered in Excel, to identify patterns in the various steps of cleaning and materials utilized. We shared preliminary findings in a meeting with USAID-implementing partners in the Zika response on June 19, 2018, in San Salvador, to obtain feedback on their interpretation and discuss the implications for program design.

## Results

### Sociodemographic characteristics of study participants

The focus groups, each with three to nine participants, together with the 12 IDIs involved a total of 83 study participants. Participants were of Mestizo descent. Most of the women were homemakers (23 of 30 pregnant women and 16 of 23 women likely to become pregnant). Male partners of pregnant women included six agricultural workers, three food vendors, three students; the rest were artisans, a video and graphic designer and an owner of an Internet café. There were no substantial differences in participants’ characteristics between the two study sites. Table 1 summarizes participants’ demographics.

**Table 1:** Demographic description of study participants

Characteristic	Pregnant women	Male partners of pregnant women	Women Likely to become pregnant
N (Total = 83 ^**a**^)	30	26	23
Age (years)			
Mean	25	27.7	24.2
Median	23	26	25
Range	18–33	19–56	18–32
Education (years)			
Mean	7.5	9.3	10.8
Median	7.4	10	11
Range	0–14	0–17	5–17
Number of children			
Mean	1.2	0.8	1
Median	1	0.5	1
Range	0–3	0–5	0–3

^a^ Two participants did not report demographic data because they joined the study late, and additional data for two other participants were lost

### The range of elicited Zika prevention behaviors in the community

During the individual free elicitation exercise, participants mentioned over 200 actions that people in their community took to avoid Zika. On average, participants mentioned five (range zero to 12) preventive actions. There were no substantial differences between the two study sites, nor between men and women participants, except for condom use, only mentioned by the men. We consolidated similar wordings of the same action into a single common wording. Table 2 shows the first 34 of a total of 43 actions ordered by descending frequency.

**Table 2:** Frequency of Zika prevention actions elicited from participants ^a^

Action mentioned	Frequency
Pick up/eliminate containers/other items that collect water	47
Keep the house and surrounding areas clean and orderly	40
Use a mosquito net	29
Clean/wash washbasins/barrels/containers with bleach	28
Clean/wash washbasins/barrels/containers (with no mention of bleach)	26
Use Abate or the “new powder” (larvicide)	24
Practice general hygiene	22
Fumigate	18
Empty/throw out water that accumulates in containers	18
Dispose of garbage (throwing it away or burning it)	17
Cover water (storage) containers	17
Receive information/give and attend talks about Zika	11
Carry out campaigns in the community	11
Clean rain gutters, spouts, channels, or the roof of the house	10
Use the *Untadita* method with containers	10
Turn containers that accumulate water over/face down	9
Clean or purify the water	7
Make smoke to drive away mosquitoes	7
Change the water in containers	6
Eliminate water puddles	5
Use a condom	5
Have promoters inspect the home	4
Use fish in the washbasin	4
Fill tires with dirt	4
Use repellent on the skin	4
Recycle things that could become breeding places	3
Maintain (hot-cold) balance in the body	3
Go in for a prenatal visit	3
Use insecticide spray	3
Practice abstinence	2
Use contraceptives to prevent pregnancy during Zika	2
Receive vaccinations from the health promoters	1
Use clothing with long sleeves during pregnancy	1
Cover toilets	1

^a^ Ten participants from IDIs and 69 from Focus Groups

Actions related to cleaning were among the most frequently mentioned. Cleaning of water storage containers was the most mentioned practice (64 mentions) when grouping cleaning modalities: cleaning without bleach (26 mentions), cleaning with bleach (28 mentions), and the *untadita* method (10 mentions) (Table 2). Moreover, the frequency of mentions of bleach utilization, either explicitly or as part of the *untadita* method, indicated the importance of cleaning water storage containers and the importance of bleach in the container cleaning process, strong community norms around this practice, and the idea that bleach might be affecting *Ae. aegypti* larvae or eggs in some manner. After water storage container cleaning, the most commonly mentioned prevention action was the clean-up of containers that accumulate or hold water (47 mentions). The third most commonly mentioned action was general cleaning and tidying the house (40 mentions, Table 2). In their unprompted explanations of general cleaning and tidying practices, many of the latter participants associated *Ae. aegypti* with dark and dirty places where adult mosquitoes and other pests were found, including in vegetation around the house. Some felt that if they lived in a clean house, they no longer needed to adopt other Zika prevention behaviors, such as use of condoms during pregnancy and skin repellent use. Furthermore, they recommended bleach for mopping the floor or pouring into water storage containers with an understanding that its odor would repel adult mosquitoes.

Another key finding from this exercise was that participants mentioned less effective prevention methods more frequently than more effective methods. The use of a mosquito net, which is less effective for a largely day-biting mosquito, was mentioned more frequently than water storage container cleaning methods; similarly, general hygiene, water purification, and the elimination of puddles were mentioned more frequently than use of mosquito repellent on the skin and use of condoms.

### Perceived effectiveness and feasibility of cleaning water storage containers

Figure 1 highlights the averaged ratings for effectiveness and feasibility of the 18 studied behaviors for preventing Zika, as a result of dialogue and consensus among the focus group participants. There were no substantial differences between the two study sites, nor between men and women participants, except for condom use which women generally thought it was very difficult to practice during pregnancy, while some men found it acceptable in light of Zika.

Measures used to clean water storage containers were rated as both highly effective and highly feasible. Participants explained that households already held existing practices of cleaning their water storage containers as part of general cleanliness. Participants perceived that communities readily had the necessary tools—such as bleach, detergent, brushes and sponges—to perform the action. Beyond the effort and time to clean the containers, the perceived cost was minimal; only some mentioned that if people did not have money for detergent or bleach, they would have to clean with water only. Most participants mentioned that the use of bleach was commonplace among households, but at the same time they revealed their misunderstandings regarding its strength: *“we use bleach to kill larvae, bacteria, things ... but only in water we don’t use to drink.*” (Non-pregnant Woman, rural site, 19GFMN1GUMR).

It is important to note that communities used two different words in Spanish for bleach, *cloro* and *lejía*. The meaning of these two words varied among participants, but a general difference that emerged was that *cloro* was a less concentrated bleach used solely for the purification of drinking water, while *lejía* was a more concentrated form of bleach used for cleaning laundry, floors, grime from plates, and washbasins. Many participants noted that *lejía* was readily available in local stores, while *cloro* was generally dispensed by local health centers. Despite this perceived difference in the two products, a review by the study team of bleach brands in local stores indicated that many commercial products contained both words on their labels.

A majority of participants agreed that cleaning the washbasin was a highly effective measure for reducing the risk from mosquitoes, as long as everyone in the neighborhood did it:

*“If we don’t all do the same thing in our homes, this not only affects us but everyone else in the community. My neighbor might clean his washbasin, but if I don’t do it and I expect him to do it, in a way I am a threat to the health of my neighbors. So that is why it is important.”*—Man, rural site, 19ENPM1GUSM

Many participants indicated that cleaning the washbasin became even more critical during prior dengue epidemics in the country. Some even mentioned that they learned to clean the *pila* at school. It was during these epidemics, that the *untadita* method was introduced according to participants’ memory. However, many participants erroneously thought that the *untadita* was effective for removing the larvae of the mosquito rather than removing the eggs. The cleaning method varied depending on the amount of dirt and mildew in the washbasin, the size of the washbasin, and local custom. For that reason, the reported time required to effectively clean a washbasin varied greatly between 10 min to 2 h.

Water storage containers other than the cement washbasin, such as barrels and drums, were less common in participants’ households. When families did have them, these were commonly located in the bathroom. Due to the more frequent use of the water stored in these containers, participants stated that they were cleaned more frequently, but not as thoroughly.

*“For example, I wash the one in the bathroom the same way [as a washbasin]. But I wash that one more often because the water runs out faster. I don't wash it with bleach but just with the broom and that's it, because the water is used up so fast [that it's not necessary].”* —Pregnant Woman, urban site, 16GFME1ILBLM

### The effect of seasonality on frequency of water storage container cleaning

Because cleaning requires much water, this behavior was performed less frequently during the Salvadoran summer between November and April, when water is less available. During this dry period, the frequency of cleaning the washbasin varied between every three to 15 days, according to how quickly the water was consumed and when the local water distribution day occurred. Participants of either gender and study site mentioned that during their winter, when rain is more frequent, it was common for cleaning to take place twice a week or when the water arrived at their home. If water arrived too soon or off-schedule, it was easy to miss the opportunity to clean the washbasin. Participants also reported that the water that arrived at households during the rainy season was cloudier. Washbasins, barrels, and drums were cleaned more frequently due to the perception that cloudy water was more prone to produce a green slime as well as contain more larvae brought in directly by the rainwater.

### Gender roles and water storage container cleaning

Both women and men participants in either study site discussed how cleaning the washbasin and the water barrels was considered to be the responsibility of the homemaker, who typically was a woman. One man from the rural site said that in his community, men’s prevailing mentality was that “*the broom is for the women”* and that a man caught doing a woman’s chores would be ridiculed or bullied. Pregnant women in a focus group said:

Participant 1: *“*We *are the ones who do the housework, so there are men who say: ah, I am not a woman.”* Participant 2: *“That is why I obtained* you*, they say …*” [laughs by all] --Pregnant women, urban site, 17GFME1IBMR3

Although some men said that they did clean the washbasins, many women reported that men do not do the job well because they do it too quickly. All participants said that it was common for women to clean washbasins until late in their pregnancy, whereupon the responsibility shifted to an adolescent, a mother-in-law, or, in rare cases, their male partner. Some male participants felt that the use of bleach could be dangerous because it could make the washbasin floor slippery for a pregnant woman. A few female participants stated that the physical position pregnant women take while cleaning a washbasin might hurt the baby’s spinal cord, and a man from the urban site said that pregnant women should not be exposed to bleach because breathing its fumes could damage the baby’s lungs.

During the IDIs, male partners of pregnant women described gender roles in some detail. Some said that the men could not clean the water storage containers because they worked long hours outside the home and came home tired.

*“It is worse when one’s wife is pregnant, because the man feels that more responsibilities are coming, and strives to work even more, therefore he has even less free time. This causes the [pregnant] wife’s housework to also increase, because during the time that the husband is not at home, the housework falls on her.”* —Man, rural site, 19ENPM1GUBA

Other men participants said that conserving water was extremely important, and container cleaning required a lot of water, thus the ideal moment for this task was when the water arrived at their home. Only at that moment could the household afford to empty the container and use running water to clean it rather than spend its stored water on cleaning; but men tended to not be at home at that time, so women were expected to do that task. However, several men said that they help with cleaning the water storage containers when they are at home resting from work, during their partner’s pregnancy and beyond, because as men, they always worry for the health of both the mother and the baby, *“and this is the best way to eliminate the mosquito; if you just drain the water and do not scrub with a brush, they will not die, mind you!”* (Man, rural site, 18ENPM1GUMR).

Men who stated that they cleaned the washbasin, attributed their behavior to their upbringing since a young age. Cleaning became a custom for them, despite working long hours outside the home, because their families did not associate the action with the shame related to traditional gender roles. However, the majority of men did not report such experiences during childhood. Many men and women acknowledged that most men are brought up with an exaggerated sense of manliness or *machismo* as the norm. Finally, male participants mentioned that unlike organized community clean-ups in which men publicly took collective action with their peers, washbasin cleaning occurred privately in the home. This did not generate the same enthusiasm because it was perceived as a part of regular household chores carried out by the women.

### Assessing skills to reduce *Ae. aegypti* eggs Vis-à-Vis the process of cleaning the washbasin

**Fig. 2:**
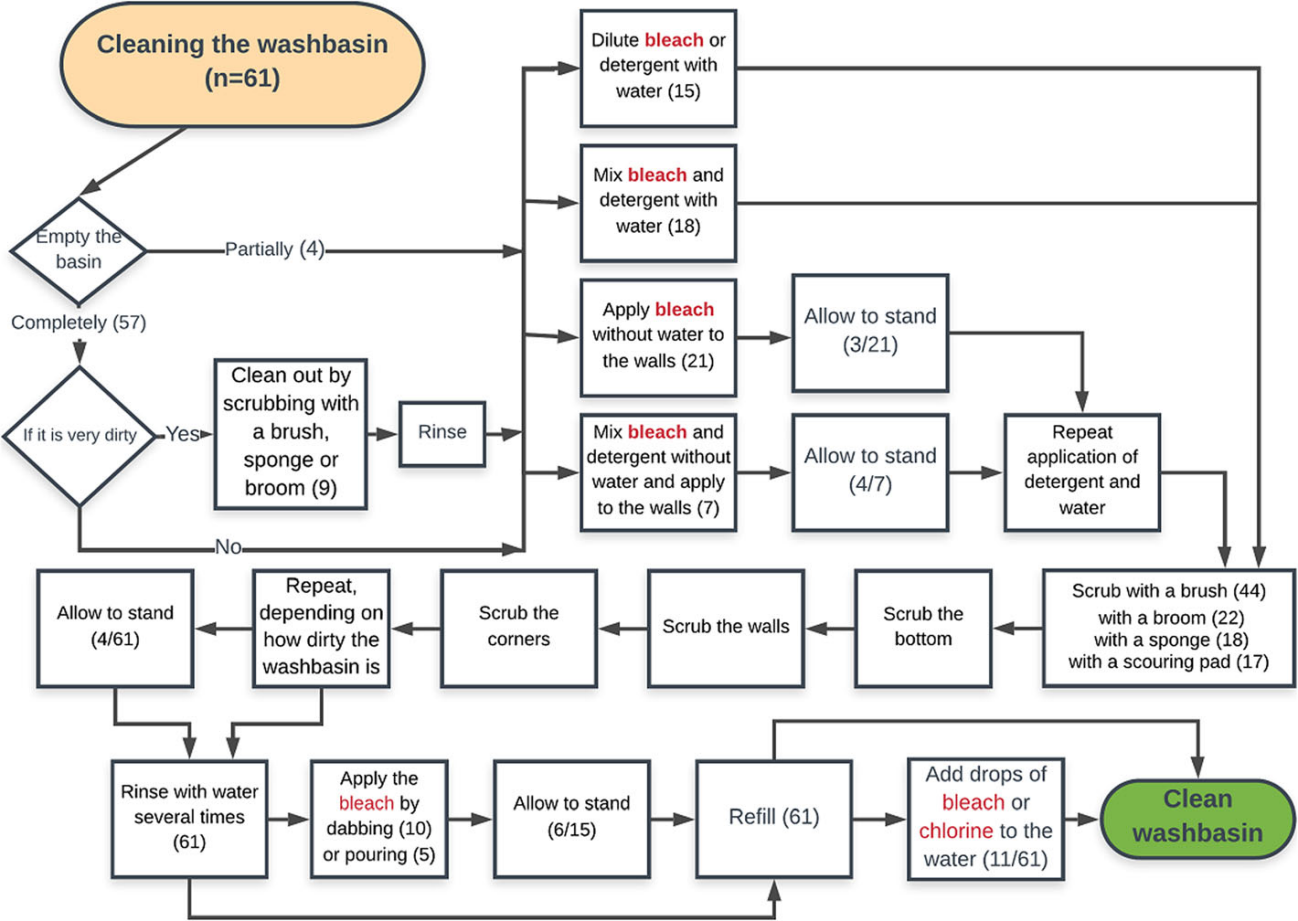
Washbasin cleaning process across 11 focus groups. Data available from 61 of 71 focus group participants; four participants could not write, and six others left early

The flowchart in Fig. 2 shows the washbasin cleaning modalities as described by the participants in writing and performed in the simulations.

There were no substantial differences in the findings between the two study sites, nor between men and women participants who provided comparable detail. Community members used a range of approaches to clean their washbasins. Nine participants reported a pre-scrub without bleach or detergent if the washbasin was very dirty, and all reported use of bleach before the washbasin was scrubbed. However, over half of the participants stated that bleach was diluted in the water at the bottom of the washbasin to dissolve a green slime that often formed there and on the walls. The purpose of wall scrubbing was to remove the green slime and dislodge the mosquito eggs. The water-bleach dilution at the bottom was believed to kill any dislodged eggs that fell into it as well as any larvae that were in the water before the scrubbing process started.

Twenty-eight participants referred to dabbing undiluted bleach, with or without detergent, as recommended by the *untadita* method. Men appeared more aware of the *untadita* and indicated they learned the technique through mass media a few years prior. However, only seven of the 28 participants acknowledged the crucial step of allowing the bleach dab to stand, mentioning five to 10 min, or long enough for the bleach to dry. The rest did not report giving the bleach dab sufficient time to kill any eggs. Four participants reported waiting a variable amount of time after scrubbing, with the intention of allowing any dislodged eggs or larvae found in the water to be killed by the bleach dilution. Fifteen participants dabbed or poured undiluted bleach on to the walls after first having used bleach to scrub the walls in a prior step (Fig. 2). This second application of bleach was intended to kill mosquito eggs or larvae, purify the water upon falling in the washbasin, and leverage the strong odor of bleach to repel mosquitoes:

*“A little bit of bleach like this [poured from a small packet along the walls] when [the washbasin] is empty so [the mosquito] doesn’t leave its eggs.”* —Pregnant Woman, urban site, 17GFME1IBMR3

Finally, 11 participants completed the washbasin cleaning process by adding a few drops of bleach to the water after filling the washbasin (Fig. 2) in order to keep their water protected.

### Community intentions to practice the Zika prevention behaviors

There were no substantial differences between the two study sites, nor between men and women participants, in voting on the top three actions which their communities would be most willing to practice, shown in Table 3. The removal of containers from the yard (26 votes) and picking up garbage around the house (25 votes) received more votes than the cleaning of the washbasin (16 votes) and applying bleach to the walls of tanks/barrels (13 votes). Cleaning water storage containers, such as barrels, also received a higher number of votes (25) because that type of cleaning was considered quicker and less burdensome. This voting indicates that positive intentions around general cleaning were stronger among study participants than the specific ovicidal cleaning to control *Ae. aegypti.*

**Table 3:** Focus group participants’ votes for behaviors that community members would be most willing to practice to prevent Zika

Behavior	Total Votes ^**a**^
Empty/Eliminate containers from around the yard	26
Go to prenatal care visits	26
Clean water storage containers (barrels)	25
Use a mosquito net	25
Pick up garbage around the house	25
Cover water storage containers	17
Clean the washbasin	16
Apply chlorine to the walls of tanks / barrels	13
Community / collective cleaning operations	8
Use larvicide	7
Dispose of tires	5
Use insect repellent on the skin	2
Abstain from sexual relations	2
Use modern family planning methods	1
Use condoms during pregnancy	1
Fumigate	1
Use screens on the windows and doors of the house	0
Wear long sleeves	0

^a^ Three votes per participant; voting data available from 65 of 71 participants, as six participants left early

## Discussion

We explored key determinants of a series of preventive behaviors for Zika, and by extension, other *Aedes-*transmissible viruses, such as dengue and chikungunya, in an urban and a rural community in El Salvador. Our study sample size was adequate to achieve thematic saturation [30] and, therefore, attain information power commensurate with our study aim [31]. Our multimethod study design enabled extensive triangulation of the data.

We specifically addressed the determinants for water container cleaning because of its central role in controlling *Ae. aegypti* populations in the home and the significant focus on this preventive action by community-based arbovirus control programs. Cleaning-related tasks, such as discarding of disposable containers around the home, keeping the house tidy, covering and cleaning water storage containers, and using bleach in the process, scored high on many explored determinants, such as positive norms, perceived effectiveness, feasibility, and positive intentions, by both women and men, as fundamental to one’s well-being. At the same time, we found inadequate skills when it came to effective *Ae. aegypti* control, important environmental constraints, such as seasonal water scarcity limiting the frequency of cleaning containers, and gender-limiting social support for frequent and thorough cleaning of the containers within the household.

The association of adult *Ae. aegypti* habitats with those of other pests found in dirty and dark places, and with overgrown vegetation near the house indicates that communities may be paying more attention to the adult stage of *Ae. aegypti*, thus misdirecting their cleaning efforts away from destroying this mosquito’s larval habitats. The finding that living in a clean house renders other Zika prevention behaviors, such as use of condoms during pregnancy and skin repellent use, unnecessary is of concern, as is the proposition to mop the floor with bleach or pour bleach into water storage containers, thinking that its odor would repel adult mosquitoes. Both of these interpretations are at worst ineffective and at best inadequate measures for *Ae. aegypti* control.

Participants revealed the community’s extensive application of household bleach, throughout the simulation of water storage container cleaning (Fig. 2). Bleach was perceived as easily accessible for most community members and was regarded as highly effective by households. Some participants were aware of the term and practice of the *untadita* as a method for controlling *Ae. aegypti*, pointing to a degree of programming continuity since the 2002 dengue prevention campaigns. Despite such attentiveness, we documented a misdirected overuse of bleach during the cleaning process, and inadequate skills to use bleach as an ovicide. To start, bleach was weakened by being mixed in water. Instructions were often interpreted as pouring a certain amount of bleach directly into the water, in order to destroy the larvae or the eggs that fell in the water after being dislodged through scrubbing; however, bleach dilutions are ineffective against either [8]. Furthermore, bleach was often poured onto the walls straight from the bag, thus allowed to run straight down to the bottom, or added directly to the water after the container was refilled after cleaning. The latter was in accordance with guidance for water treatment issued by diarrheal disease prevention programs. However, for effective *Ae. Aegypti* control, bleach has to instead be purposefully dabbed on the area above the waterline where *Ae. aegypti* eggs are found. Many participants reported or demonstrated a second application of bleach on the walls and the water, following scrubbing. This was an unnecessary repetition, instead of applying the bleach correctly the first time. Lastly, few participants reported enough time for the bleach to act on the eggs. These results indicate that communities could spend a shorter amount of time cleaning with less bleach and be more effective both in cleaning containers and in controlling *Ae. aegypti* by destroying mosquito eggs. Furthermore, given that cleaning washbasins took place every two to 3 days in most cases, communities were cleaning their water storage containers with more effort than required.

The cleaning behaviors that participants described also reflected information heard during previous waterborne illness and dengue prevention campaigns. Campaigns, including those during the Zika outbreak, emphasized that washbasins should be “cleaned,” which can be done by a *limpieza* or a *lavado* meaning a “cleaning” or a “washing,” respectively. This was in agreement with findings from another qualitative study conducted in Guatemala [32]. As a result, participants used these terms interchangeably to describe the process of sanitizing their water containers, and not specifically for *Ae. aegypti* control. This lack of specificity in the terms used by Zika prevention campaigns might have led community members to fill knowledge gaps with their prior understandings, and as a result, dilute the effectiveness of cleaning water storage containers for mosquito control, potentially creating a false sense of security regarding Zika prevention.

Lastly, our study showed that women bear the primary responsibility for water storage container cleaning in the household, consistent with their overall power and authority in the domestic domain [33]. Their responsibility for cleaning of these containers was reinforced by three factors; (a) scarcity and intermittent supply of water forcing households to limit cleaning only when water arrives at the household [11] which usually occurs when men are out working; (b) gender norms that assign cleaning of washbasins to women [34, 35]; and (c) women’s assessment of men’s cleaning performance as suboptimal. However, we discovered that as a woman approaches late pregnancy, other family members, including male partners, may assume the role of cleaning. Despite men’s long working hours outside the home, many men reported a high willingness to provide substantial social support with cleaning, during pregnancy, as protectors of the family. This was in agreement with two other qualitative studies conducted in Guatemala and the Dominican Republic [32, 34], and it adds to the role that men can play, beyond involvement in family planning or preventing the sexual transmission of Zika during an outbreak [36].

Our study has two limitations. First, it only represented the opinions of participants from two departments in El Salvador, an urban area and a rural area. Due to financial, time, and security constraints, we did not include participants from additional departments. However, both sites were impacted by the Zika outbreak like other urban and rural Salvadoran communities. The second limitation was that the study investigated the perceptions and key factors regarding community actions and not the actual behaviors of the participants. However, the simulations and projective techniques used in the study were close proxies that enabled participants to openly share their perspectives without disclosing their personal conduct.

Our findings translate to several programmatic recommendations. Households need clarity regarding the specificity of methods to reduce *Ae. aegypti* infestation*.* Cleaning out places where adult mosquitoes often hide, such as dark or dirty corners in the home or in the underbrush outside, would not eliminate mosquitoes at their source; the focus should be on the eggs and larvae instead [37]. Instead of simply informing communities to clean their water storage containers, interventions should emphasize and actually demonstrate the places where *Ae. aegypti* lays its eggs: on the vertical walls of water containers, not in the water [32], and should recommend *dabbing*, *saturating with* bleach or *spreading* undiluted bleach onto those walls (*untar*, *empapar* or *chuponear* [14]), instead of *using*, *scattering* or *pouring* (*utilizar*, *regar,* or *echar*). Coordination between public and private sectors is required to achieve the clarity needed.

Implementing organizations should leverage the existing norm of cleaning because it is already a habit among most households. However, we recommend that stakeholders reframe the *untadita* to distinguish it from existing understandings of general hygiene and water purification. One approach could be to brand the *untadita* as a treatment (*tratamiento)* specifically to remove the *Ae. aegypti* eggs, not the larvae, to apply after people clean their washbasins [13]. This tactic would separate the *untadita* from general cleaning while still allowing it to be linked to the current habit of washbasin cleaning*.* This would be similar to a behavioral economics approach such as ‘piggybacking’ [38]. For example, success has been shown in inducing individuals to floss more when it is coupled with teeth brushing [39]. Furthermore, health communication activities should emphasize that the *Ae. aegypti* mosquito differs from other pests that are considered dirty and a result of poor hygiene. Interventions should make it clear that while actions of general cleanliness and hygiene are excellent for overall health, they are ineffective for preventing arbovirus-related illnesses.

Companies that supply bleach to consumers are important actors within the private sector and could play a larger role in *Ae. aegypti* control efforts. Although not reported in the results, our team took photos of common packaging of local bleach brands found in supermarkets and convenience stores in El Salvador. A minority of the national brands made references to mosquito prevention with little or no explanation. Only one label out of five noted that bleach could be used for the *untadita* method; however, it did not provide instructions on how to correctly perform the technique. Such minimal information may lead consumers to make misguided assumptions about how bleach works to prevent mosquito infestation. Furthermore, one large multinational bleach supplier that operated in El Salvador posted recommendations on their corporate responsibility web page that differ greatly from the *untadita*: *“The data suggests that supplemental cleaning with bleach or treating water in containers that can’t be emptied may reduce the attractiveness of these containers as breeding sites. This supplemental step is now included in our public education materials.” (*Corporate Responsibility, The Clorox Company [35]). Harmonizing the messaging on bleach packaging with respected health authorities, such as Ministries of Health, could serve as an excellent touchpoint to reinforce the proper use of bleach for reducing *Ae. aegypti* at the household level. At the very least, efforts to harmonize such messaging would help reduce the inconsistencies that we observed about bleach use for Zika prevention in this study.

The infrequent cleaning of water storage containers during the dry season, when the water supply is extremely scarce in some communities, represents another unresolved problem. Partial application of the *untadita* in the container, above the water level, without emptying the water out has been considered as a temporary solution in past campaigns [13]. This option should be re-examined by health authorities and implementing organizations.

There is potential to sustain the reported high feasibility of such behaviors in households even during pregnancy. Families would do well to discuss how washbasins will be effectively and consistently cleaned to remove *Ae. aegypti* eggs at least throughout pregnancy. Implementing organizations should encourage such discussions with men. Detection of pregnancy, especially among couples expecting a first child, may be enough of a disruption of the *status quo* for a new behavior to be adopted [40].

Finally, our data indicate that health communication activities should more effectively draw a distinction between preventive behaviors targeting *Ae. aegypti* versus the Anopheline vectors of malaria. The use of mosquito nets was rated high in norms (Table 2), effectiveness and feasibility (Fig. 1), as well as community intentions for future practice for the prevention of Zika (Table 3). There is a role for nets to play when people nap during the daytime, especially pregnant women, and to prevent *Aedes* mosquitoes from becoming infected with an arbovirus from viremic individuals while lying in bed and transmitting it to the virus-free. In addition, there is some evidence to suggest that nets lower the risk of dengue or the number of dengue-infected mosquitoes or the *Aedes* infestation indices in some instances [41–44], but not in others [43, 45]. That said, the protective role of nets is much larger, and easier to measure against *Anopheles* than *Aedes* because *Anopheles* bite primarily at night. In contrast, the majority of *Ae. aegypti* biting activity is during the day [7]. Communities likely do not distinguish between the behavior of these two vectors, and concurrent malaria prevention campaigns correctly promote the use of mosquito nets. Indeed El Salvador is the first Central American country on the way to achieving official malaria-free certification by the World Health Organization [46]. As a result, communities may have developed a false sense of security by focusing their efforts on this less effective prevention method when it comes to arboviruses.

## Conclusions

Behavior change programs for the prevention of Zika and other arboviruses need to improve community members’ mosquito egg destruction skills rather than perpetuate the promotion of non-specific cleaning in and around the home as an effective measure. Egg elimination must be clearly identified as the objective of water storage container maintenance and programs should highlight the effective techniques to achieve this goal. In addition, programs must build the skills of family members who support pregnant women to maintain the frequency of effective egg destruction in all water storage containers of the home. Our findings can help Zika and other arbovirus disease prevention programs sharpen their behavioral focus and improve their health communication efforts in El Salvador and similar settings in the LAC region.

## Supplementary information

**Additional file 1.** PUBH-D-20-01205 Focus Group Guide 4.15.18 Spanish.docx

**Additional file 2.** PUBH-D-20-01205 Focus Group Guide 4.15.18 English translation.docx

**Additional file 3.** PUBH-D-20-01205 Interview Guide men 4.15.18 Spanish.docx

**Additional file 4.** PUBH-D-20-01205 Interview Guide men 4.15.18 English translation.docx

**Additional file 5.** PUBH-D-20-01205 Codebook May 2018 Spanish.docx

**Additional file 6.** PUBH-D-20-01205 Codebook May 2018 English translation.docx

**Additional file 7.** PUBH-D-20-01205 COREQ list.docx

## Data Availability

The data that support the findings of this study are not publicly available because we did not ask participants to consent to raw data sharing outside of the research team. Public sharing of the data could compromise research participant consent.

## Abbreviations

*Ae. aegypti*: *Aedes aegypti*
USAID: The United States Agency for International Development
IDIs: In-depth interviews
UNICEF: United Nations Children’s Fund
MCDI: Medical Care Development International
CCP: Johns Hopkins Center for Communication Programs

## Footnotes

1 Zika is also sexually transmitted.

2 The CHANGE Project (1998–2003) was a cooperative agreement between USAID and the Academy for Educational Development with its subcontractor, the Manoff Group, to provide leadership for behavior change innovation and state-of-the-art tools and strategies.
