# Supplementary material for: A qualitative study of community perspectives surrounding cleaning practices in the context of Zika prevention in El Salvador: implications for community-based Aedes aegypti control
Source: BMC Public Health. 2020 Sep 11;20:1385. doi: 10.1186/s12889-020-09370-5 (PMC7488301; doi:10.1186/s12889-020-09370-5)
Supplement: Supplementary file 1 — Additional file 1. PUBH-D-20-01205 Focus Group Guide 4.15.18 Spanish.docx [file 12889_2020_9370_MOESM1_ESM.docx]

Guía de preguntas Grupo Focal

*Antes de que empiece el grupo focal*

**1. Confirmar elegibilidad y obtenga el consentimiento informado oral individualmente.**

**2. Administre ejercicio de Elicitación Libre a cada participante individualmente a medida que llegan y mientras esperan que comience la discusión grupal**

**3. Introducción del grupo focal**

1. Ustedes nos han dado una lista de acciones que las personas de esta comunidad hace para evitar el Zika - gracias. Ahora vamos a platicar más sobre este tema.
2. Durante nuestra conversación, no hay respuestas equivocadas o correctas. Esto no es una charla, ni una capacitación. Venimos a escuchar las opiniones de la comunidad. Por eso, todas las respuestas son valiosas.
3. Reglas del grupo:
   - Queremos escuchar a todos.
   - Es importante que respetemos las opiniones de todos.
   - Colocar el celular en vibrador para que no interrumpa la dinámica de la discusión
4. Rompehielo
   - Antes de empezar queremos conocernos mejor. Vamos a pasar rápido por el salón individualmente y cada uno se presentará diciendo el nombre. Luego mencionará ¡qué le molesta más sobre el mosquito!
5. **Nivel de Preocupación por Zika**
   1. **¿En general qué problemas de salud preocupan a las personas?**
   2. En su comunidad **¿ Qué se ha oído del Zika?**
   3. Cuando vino el Zika a El Salvador, **¿Cómo reaccionó su comunidad?**
   4. **¿Y qué pensaron de Zika en relación a los otros problemas de salud, como** (*decir algunos problemas que han mencionado*)**? Y ahora ¿qué piensa su comunidad sobre Zika?”**
6. **Clasificar tarjetas: Parte 1 - efectividad**
   1. Ahora vamos a hacer una dinámica y organizar en grupo estas tarjetas.
   2. *Muestre las tarjetas con los imágenes de las diferentes acciones y haga una descripción rápida de cada acción representada.*
   3. *Luego coloque la tarjeta en la mesa. Solicite a los participantes que organicen las tarjetas en tres grupos (muy efectivas, más o menos, poco efectivas) respondiendo a la siguiente pregunta:*

**-¿Cuáles de esas acciones son efectivas / funcionan mejor/ dan mejores resultados para evitar el Zika, según la opinión de su comunidad?**

- 1. *Sondear las razones del* **porqué fueron ubicadas en cada categoría** *(Muy efectivas, más o menos, poco efectivas):*

*Ejemplos de sondeos:*

1. *Para que la medida funcione*, ¿De qué depende? *De la frecuencia para realizarla, tiempo, dinero, actitud, conocimiento, costumbres.*
2. *Para que la medida funcione,* ¿De quién depende? *De las personas la comunidad, los líderes de la comunidad, las autoridades.*
3. ¿Qué recursos se necesitan?
4. **Clasificar tarjetas: Parte 2 - factibilidad**
   1. Ahora vamos a agrupar las tarjetas en otros tres grupos *( Muchas posibilidades, más o menos, pocas posibilidades) respondiendo a la siguiente pregunta:*

**- ¿Qué posibilidades tienen las personas en su comunidad de poner en práctica esas medidas?**

- 1. *Sondear las razones del* **porqué fueron ubicadas en cada categoría** *( Muchas posibilidades, más o menos, pocas posibilidades)*:

*Ejemplos de sondeos:*

- - 1. Cantidad de pasos *necesarios para hacer la acción*
    2. *Lo* fácil o difícil *que es hacer los pasos*
    3. *Lo* fácil o difícil *que es organizar/planificar la acción (por ejemplo tener que encontrar alguien que cuida a los niños, tener que hacer citas, etc.)*
    4. El tiempo *requisito para hacer la acción (tener que hacerlo sólo una vez o se repite, etc.)*
    5. *Lo* fácil o difícil *que es conseguir/obtener lo necesario para hacer la acción*
    6. Costo/precio *de los insumos/materiales*
    7. *Hasta qué punto la acción es una* norma *en la comunidad*
    8. *Qué dicta* la cultura *acerca de la acción*

1. **Contrastar las tarjetas: Parte 3**
   1. Ahora que hemos revisado todas las acciones una por una, quisiera que reflexionáramos sobre la organización de las tarjetas:

-**¿Porqué algunas son posibles hacerlas, pero no funcionan para la prevención del Zika? y al**

**revés.**

**-¿Cómo se puede ayudar a las personas para que la medida de prevención de Zika sea posible realizarla?**

-**¿Cómo se puede ayudar a las personas para que la medida de prevención de Zika funcione mejor?**

- 1. Pasen enfrente a ver las acciones en sus grupos de efectividad y posibilidad

**-¿hay una acción que quieren recolocar?**

1. **Votos** Ahora voy a darles tres calcomanías cada uno. Por favor,

-**elija** (colocar sticker) **las tres acciones que las personas de su comunidad estarían más dispuestas de hacer para evitar el Zika**

**9. Limpieza simulada de un recipiente de almacenamiento de agua**

1. Acabamos de hablar sobre muchas acciones para prevenir el Zika, incluyendo la limpieza de recipientes para almacenar agua **¿Cuál recipiente es el más utilizado en esta comunidad?**
2. Ahora vamos a hacer unas actividades acerca de cómo las personas en su comunidad limpian las pilas/barriles de agua.

**9.1 Recetas individuales de la limpieza**

1. *Dar a cada participante una hoja doblada de papel y marcadores*
2. Cada uno de ustedes individualmente, van a escribir/dibujar en detalle cómo se limpia una pila o un barríl de agua en su comunidad.
   - Al lado izquierda de la hoja, **identifiquen los materiales/insumos que se necesita**.
   - Al lado derecha de la hoja, **describen paso por paso los pasos que haría la gente en su comunidad para hacer la limpieza**.
3. *Después de la actividad recoger todos los marcadores y agradecer a los participantes.*

**9.2 Actividad para el grupo entero:**

1. Ahora vamos a trabajar todos juntos. Primero necesitamos **un voluntario** para mostrar al grupo sobre cómo las personas limpian el recipiente de almacenamiento de agua en su comunidad.
2. *Una vez que un voluntario haya sido seleccionado, enseñar el contenedor plástico de modelaje y preguntar:* Como grupo, imaginen que este contenedor de agua representa el contenedor más común en los hogares de sus vecinos: Es un(a): _______[*inserta el contenedor que ellos mencionaron es el más común en el área*]____.
3. *Preguntar al voluntario* **¿Cuáles son los materiales que necesitarían?** *Entonces sacar los materiales nombrados.*
4. *Después, pedir al voluntario a* **demostrar paso por paso cómo se limpia el recipiente, usando los materiales que mencionó**.
5. *Solicitar al resto del grupo que pongan atención y observen . El voluntario puede ver de nuevo, los pasos que detalló en su hoja.*
6. *Después que el voluntario haya mostrado los pasos, agradecer su participación y si los demás dicen que en su comunidad lo hacen diferente, preguntar* **¿cómo lo hacen?** *Solicitar que alguien pase al frente a demostrar los pasos diferentes.*
7. *Sondeo final*
   - - 1. **Cuando es verano o invierno, ¿cambia la forma de limpiar?**
       2. Durante la emergencia de Zika en el 2016, **¿cómo hicieron las personas de la comunidad las limpiezas de sus pilas? ¿Cambiaron la forma de limpiar las pilas? ¿Y ahora, que piensan que están haciendo?**
8. *Durante la actividad, un miembro del equipo tomará fotografías del proceso aplicado por el voluntario (sin tomar fotos de las caras de los participantes).*
9. *Recoger las hojas de los particpantes.*
10. **Género:**
11. **¿Quién en el hogar generalmente realiza la limpieza los recipientes de agua?**

*Sondeos:*

1. **¿Cuál es el papel de la mujer? ¿Del hombre?**

2. **¿Cuál es la diferencia cuando la mujer/ama de casa está embarazada?** Si una mujer embarazada va a limpiar la pila durante el embarazo, **¿hay algo que ella haría diferente?**

3. **¿Cómo se podría motivar a las personas para que participen en la limpieza de los recipientes de agua?**

*Pregunte si* **alguien tiene algo más para agregar.** *Agradezca y despida a los participantes.*

**FIN**
