# Supplementary material for: A qualitative study of community perspectives surrounding cleaning practices in the context of Zika prevention in El Salvador: implications for community-based Aedes aegypti control
Source: BMC Public Health. 2020 Sep 11;20:1385. doi: 10.1186/s12889-020-09370-5 (PMC7488301; doi:10.1186/s12889-020-09370-5)
Supplement: Supplementary file 2 — Additional file 2. PUBH-D-20-01205 Focus Group Guide 4.15.18 English translation.docx [file 12889_2020_9370_MOESM2_ESM.docx]

Focal Group Field Guide

*Before the focus group begins*

**1. Confirm eligibility and obtain informed oral consent individually**

**2. Administer Free Elicitation Exercise to each** **participant individually as they arrive and while they wait for the focus group to begin**

**3. Introduction of the focus group**

1. You have given us a list of actions that people in this community do to avoid Zika - thank you. Now let's talk more about this topic.
2. During our conversation, there are no wrong or correct answers. This isn't a talk, it's not a training. We came to hear the opinions of the community. That's why all the answers are valuable.
3. Group Rules:
   - We want to hear from everyone.
   - It is important that we respect everyone's opinions.
   - Place the cell phone on vibrator so that it does not interrupt the discussion flow
4. Icebreaker
   - Before we start we want to know each other better. We will pass quickly around the room and each one will introduce themsleves saying their name. Then will mention what bothers them the most about mosquitoes!
5. **Zika Concern Levels**
   1. **In general, what health problems do people worry about?**
   2. In your community, **what has been heard of Zika?**
   3. When Zika came to El Salvador, **how did your community** **react?**
   4. **And what did people think of Zika in relation to the other health problems,** **such as** (*say some problems that were mentioned*)**? And now what does your community think about Zika?"**
6. **Sorting cards: Part 1 - effectiveness**
   1. Now let's do a dynamic and organize these cards as a group.
   2. *Display the cards with the images* *of* *the different actions and give a quick description of each action represented.*
   3. *Then place the card on the table. Ask participants to organize the cards into three groups (very effective, more or less, ineffective) by answering the following question:*

**-Which of these actions are effective / work best / give better results to avoid Zika, according to the opinion of your community?**

- 1. *Probe on the reasons* **why they were placed in each category**  *(Very effective, more or less, ineffective):*

*Examples of probes:*

1. *For the measure to work,* what does it depend on? *On the frequency of performance, time, money, attitude, knowledge, customs.*
2. *For the measure to work,* who does it depend on? *On the people the community, the community leaders, the authorities.*
3. What resources are needed?
4. **Sorting cards: Part 2 - feasibility**
   1. Now let's group the cards in three other groups *(Very feasible, more or less, not feasible) answering the following question:*

**- How feasible is it for people in your community to implement these measures?**

- 1. *Proble on the reasons* **why they were placed in each category**  *(Very feasible, more or less, not feasible)*:

*Examples of probes:*

- - 1. Number of steps *needed to do the action*
    2. *How* easy or difficult it is to do the *steps*
    3. *How* easy or difficult it is to *organize/plan the action (for example, having to find someone to look after the children, having to make appointments, etc.)*
    4. The time *requirement to do the action (having to do it only once or it repeats, etc.)*
    5. *How* easy or hard it is to *get what you need to do the action*
    6. Cost/price *of inputs/materials*
    7. *To what extent action is a* norm  *in the community*
    8. *What* culture dictates *about the action*

1. **Contrasting Cards: Part 3**
   1. Now that we have reviewed all the actions one by one, I would like you to reflect on the sorting of the cards:

-**Why are some actions feasible to do them, but are not effective for** **Zika prevention? And vice versa.**

**-How can people be helped to make the Zika prevention action more feasible?**

**-How can people be helped to make the Zika prevention measure more effective?**

- 1. Come to the front and see the actions sorted by effectiveness and feasibility

**-Is there an action that you want to reposition?**

1. **Votes** Now I'm going to give you three stickers each. Please,

-**choose**  (sticker) **the three actions that people in your community would be more willing to do to prevent Zika**

**9.** **Simulated cleaning of a water storage container**

1. We just talked about many actions to prevent Zika, including cleaning water storage containers. **Which container is the most utilized for water storage in this community?**
2. Nows let's do some activities on how people in your community clean their pilas/water barrels.

**9.1**  **Individual**  **cleaning recipes**

1. *Give each participant a folded* sheet *of paper and markers*
2. Each of you individually, will write/draw in detail how is a pila or water barrel cleaned in your community.
   - On the left side of the sheet, **identify the materials/inputs you need**.
   - On the right side of the sheet, **describe step by step the steps people would take in their community to do the cleaning**.
3. *After the activity collect all the markers and thank the participants.*

**9.2**  **Activity for the entire group:**

1. Now let's all work together. First we need **a volunteer** to show the group how people clean the water storage container in their community.
2. *Once a volunteer has been selected, show the model* *plastic container and ask:* As a group, imagine that this container represents the most common water storage container in the homes of your neighbors: It is a: *_______[insert the container they mentioned as most common in the area]*______.
3. *Ask the volunteer* **What materials would be needed?** *Then provide the named materials.*
4. *Then, ask the volunteer to* **demonstrate step by step how the container is cleaned, using the materials you mentioned.**
5. *Ask the rest of the group to pay attention and watch.* *The volunteer can consult the steps they had detailed on their sheet if needed.*
6. *After the volunteer has demonstrated the steps, thank them for their participation, and* if *others say that* *in their*  *community* *they do it differently, ask* **how do they do it?** *Ask someone to come* forward *to demonstrate the steps that are different.*
7. *Final probing*
   - - 1. **When it's summer or winter, does the way of cleaning differ?**
       2. During the Zika emergency in 2016, **how did people in the community do the cleaning of their pilas? Did they change the way the cleaned their pilas? And how do you think people do it currently?**
8. *During the activity, a research team member will take pictures of the process applied by the volunteer (without taking pictures of the participants' faces).*
9. *Collect back the sheets of paper from the particpants.*
10. **Gender:**
11. **Who in the home usually cleanses the water containers?**

*Probes:*

1. **What is the** **role of women? Of men?**

2. **What is the difference when the** **woman/housewife is pregnant?** If a pregnant woman is going to clean the pila during her pregnancy, **is there anything she would do differently?**

3. **How** c**ould people be encouraged to participate in** **water container cleaning?**

*Ask if* **anyone has anything else to add.** *Thank and dismiss the participants.*

**End**
