# Supplementary material for: A qualitative study of community perspectives surrounding cleaning practices in the context of Zika prevention in El Salvador: implications for community-based Aedes aegypti control
Source: BMC Public Health. 2020 Sep 11;20:1385. doi: 10.1186/s12889-020-09370-5 (PMC7488301; doi:10.1186/s12889-020-09370-5)
Supplement: Supplementary file 3 — Additional file 3. PUBH-D-20-01205 Interview Guide men 4.15.18 Spanish.docx [file 12889_2020_9370_MOESM3_ESM.docx]

Guía: Entrevista Semi-estructurada hombres

**1. Confirmar elegibilidad y obtenga el consentimiento informado oral individualmente.**

**2. Comenzar con la actividad de Elicitación Libre**

1. Por favor darnos una lista que describa lo que la gente hace en esta comunidad para evitar el Zika.
2. *Anote todas las acciones en el orden en que el participante las menciona. Asegúrese que el participante haya mencionado todas las acciones que se le hayan ocurrido.*
3. **Nivel de Preocupación por el Zika**
4. Usted nos ha dado una lista de acciones que la gente en su comunidad hace para evitar el Zika ¡Gracias! Ahora vamos a conversar un poco más sobre este tema.
   1. **¿Cuándo fue que la gente en su comunidad comenzó a preocuparse por el Zika? ¿De qué se preocupaban las personas?**
   2. *Explore los motivos de preocupación entre los hombres*
   3. *Sondeos*
5. De acuerdo a los hombres en su comunidad, ¿qué tan importante era el Zika cuando recién apareció?
6. ¿Y cómo era para los hombres que tenían una pareja embarazada?
7. De acuerdo a los hombres en su comunidad, si comparamos como era antes, ¿qué tan importante es el Zika actualmente?
8. ¿Y cómo es para los hombres que tienen una pareja embarazada?

**2. Eliminar el agua que se acumula en recipientes alrededor de la casa**

1. *Entregue al participante la tarjeta que muestra a una familia sacando el agua de recipientes alrededor de la casa.*
2. **¿Qué cree usted que piensan los hombres de su comunidad acerca de este comportamiento?**
3. **¿Qué papel juegan los hombres en esta acción?**
4. **¿Qué es lo que influye para que los hombres tengan o no tengan un papel activo en vaciar y eliminar recipientes que acumulan agua alrededor del hogar?**
5. **Cuando un hombre tiene una pareja embarazada, ¿cómo cambia el papel del hombre acerca de esta acción?**

**3. Limpiar los recipientes de almacenamiento de agua**

1. *Entregue al participante la tarjeta que muestra a una persona cepillando las paredes de un barril de agua.*
2. **¿Qué cree usted que piensan los hombres de su comunidad acerca de realizar esta acción?**
3. **¿Qué papel juegan los hombres? ¿Típicamente, quien hace esa actividad en la familia?**
4. **Cuando un hombre tiene una pareja embarazada, ¿cómo cambia el papel del hombre acerca de esta actividad?**
5. **¿Qué es lo que influye en que los hombres tengan o no tengan un papel activo en la limpieza de los recipientes de almacenamiento de agua?**
6. **Uso de condones durante el embarazo**
7. *Entregue al participante la tarjeta que muestra a una pareja embarazada con un condón a mano.*
8. **Aquí hay una mujer** *[pida un nombre*] **y su compañero** [*pida un nombre*]. **¿Qué ve usted en esta imagen?**
9. **¿Qué cree usted que está pensando el hombre de la mujer?** Sea específico y hable como si usted fuera ella.
10. **¿Qué cree usted que está pensando la mujer del hombre?** Sea específico y hable como si usted fuera él.
11. **¿Cómo cree usted que fue la conversación que tuvo la pareja cuando hablaron por primera vez sobre el uso del condón durante el embarazo?**
12. **¿Quién cree usted que comenzó la conversación?**
13. **¿Qué afectará que ellos usen o no el condón?**
14. **Imagínese que ahora ya han pasado dos años y que el Zika todavía está presente en la comunidad.**
    1. **¿Cómo cree usted que el Zika influiría si** [el nombre de la mujer y el nombre del hombre] **quieren retrasar o evitar la llegada de otro bebé?**
    2. **¿Qué cree usted que piensan los hombres de su comunidad acerca de la planificación familiar para evitar embarazos con problemas relacionados con el Zika?**
15. **Buscar servicios de atención prenatal como pareja**
16. *Entregue al participante la tarjeta que muestra a una pareja en consejería de atención prenatal.*
17. **Aquí hay una mujer embarazada** [el nombre de la mujer] **y su compañero** [el nombre del hombre]. **¿Qué ve usted en esta imagen? ¿Cómo se relaciona lo que ve en esta tarjeta con el Zika?**
18. **¿Qué cree usted que está pensando** [el nombre de la mujer]**? ¿Cómo se siente ella?** Sea específico y hable como si usted fuera ella.
19. **¿Qué cree usted que está pensando** [el nombre del hombre]**?** **¿Cómo se siente él?** Sea específico y hable como si usted fuera él.
20. **¿Qué tan común es para un hombre acompañar a su pareja embarazada a las visitas de atención prenatal?**
21. **¿Qué afectará que los hombres jueguen o no un rol activo durante las citas de atención prenatal de su pareja?**
22. **¿Cómo es que el Zika afectó, si de alguna forma lo hizo, las parejas que esperan un bebé con respecto a las citas de atención prenatal?**
23. *Pregunte al participante* **si hay algo más que le gustaría agregar.** Agradézcale por su tiempo.

**FIN**
