# Supplementary material for: A qualitative study of community perspectives surrounding cleaning practices in the context of Zika prevention in El Salvador: implications for community-based Aedes aegypti control
Source: BMC Public Health. 2020 Sep 11;20:1385. doi: 10.1186/s12889-020-09370-5 (PMC7488301; doi:10.1186/s12889-020-09370-5)
Supplement: Supplementary file 4 — Additional file 4. PUBH-D-20-01205 Interview Guide men 4.15.18 English translation.docx [file 12889_2020_9370_MOESM4_ESM.docx]

Semi-structured Interview Guide with men:

**1. Confirm eligibility** **and obtain informed oral consent individually.**

**2. Start with the Free** **Elicitation Exercise**

1. Please give us a list describing what people do in this community to avoid Zika.
2. *Write down all actions in the order in which the participant mentions them. Make sure the participant has mentioned all the actions that have occurred to him.*
3. **Zika Concern Levels**
4. You've given us a list of actions people in your community do to avoid Zika. Thank you! Now let's talk a little more about this issue.
   1. **When did people in your community start worrying about Zika? What were people worried about?**
   2. *Explore concerns among men*
   3. *Probes*
5. According to the men in your community, how important was Zika when it first appeared?
6. And what was it like for men who had a pregnant partner?
7. According to the men in your community, if we compare as it was before, how important is Zika today?
8. And what's it like for men who have a pregnant partner?

**2. Remove rainwater that builds up in containers around the house**

1. *Give the participant the card showing a family by pulling the water out of containers around the house.*
2. **What do you think men in your community think about this behavior?**
3. **What role do men play in this action?**
4. **What influences men to have or not have an active role in emptying and removing containers that accumulate water around the home?**
5. **When a man has a pregnant partner, how does his role change regarding this action?**

**3. Clean water storage containers**

1. *Give the participant the card showing a person brushing the walls of a barrel.*
2. **What do you think the men in your community think about doing this?**
3. **What role do men play? Typically, who does that activity in the family?**
4. **When a man has a pregnant partner, how does his role change regarding this action?**
5. **What influences men or not to play an active role in cleaning water storage containers?**
6. **Use condoms during pregnancy**
7. *Give the participant the card showing a pregnant couple holding a* condom in their hand*.*
8. **Here is a woman** *[ask for a name*] and **her companion** [*ask for a name*]. **What do you see in this picture?**
9. **What do you think the man is thinking of the woman?** Be specific and talk like you're her.
10. **What do you think the woman is thinking of the man?** Be specific and talk like you're him.
11. **How do you think the couple's conversation went when they first talked about condom use during pregnancy?**
12. **Who do you think started the conversation?**
13. **What will affect them in using condoms or not?**
14. **Imagine that it's been two years now and that Zika is still present in the community.**
    1. **How do you think Zika would influence if** [the woman's name and the man's name] **want to delay or prevent the arrival of another baby?**
    2. **What do you think men in your community think about family planning to avoid pregnancies because of Zika-related issues?**
15. **Seek prenatal care services together as a couple**
16. *Give the participant the card showing a couple in prenatal care counseling.*
17. **Here is a** **pregnant woman** [the woman's name] **and her companion** [the man's name]**.** **What do you see in this image? How does what you see on this card relate to** **Zika?**
18. **What do you think** [the woman's name] **is thinking?** **How does she feel?** Be specific and talk like you're her.
19. **What do you think** [the man's name] **is thinking? How does he feel?** Be specific and talk like you're him.
20. **How common is it for a man to accompany his pregnant partner to prenatal care visits?**
21. **What will affect men in playing an active role or not during their partner's prenatal care visits?**
22. **How did Zika affect, if it did in any way, pregnant couples with regard to prenatal care visits?**
23. *Ask the participant* **if there is anything else, they would like to add.** Thank him for his time.

**End**
