# Supplementary material for: A qualitative study of community perspectives surrounding cleaning practices in the context of Zika prevention in El Salvador: implications for community-based Aedes aegypti control
Source: BMC Public Health. 2020 Sep 11;20:1385. doi: 10.1186/s12889-020-09370-5 (PMC7488301; doi:10.1186/s12889-020-09370-5)
Supplement: Supplementary file 5 — Additional file 5. PUBH-D-20-01205 Codebook May 2018 Spanish.docx [file 12889_2020_9370_MOESM5_ESM.docx]

**Grupos de códigos**

**Códigos**

Abastecimiento de agua

**Explicación del código**

Frecuencia y cantidad de agua a las que las familias tienen acceso, ya sea por medio del servicio

municipal, compra o recoger agua de lluvia o río Percepción de efectividad de los métodos de

planificación familiar en la prevención del zika, según agrupamiento de láminas

Discusión sobre contraste entre efectividad y

factibilidad de los métodos de planificación familiar según agrupamiento de láminas

Percepción de factibilidad de los métodos de

planificación familiar en la prevención del zika, según agrupamiento de láminas

Uso de métodos de planificación familiar

Percepción de efectividad del control prenatal para la prevención del zika

Discusión sobre contraste entre efectividad y

factibilidad del control prenatal según agrupamiento de láminas

Percepción de factibilidad del control prenatal en la

prevención del zika, según agrupamiento de láminas Conocimientos, actitudes y prácticas respecto al control prenatal

Aplicación de cloro/lejía directamente en la pared de

la pila o recipiente de agua (untadita y otras formas de aplicación)

Aplicación de cloro/lejía directamente de la bolsa a las

paredes de la pila o recipiente de agua

E. Métodos de planificación familiar

Anticonceptivos/Planificación familiar

efectividad

Anticonceptivos/Planificación familiar

efectividad/factibilidad

Anticonceptivos/Planificación familiar

factibilidad

Anticonceptivos/Planificación familiar

Asistir atención prenatal efectividad

C. Asistir atención prenatal

Asistir atención prenatal

efectividad/factibilidad

Asistir atención prenatal factibilidad

Asistir atención prenatal uso

Uso de lejía

Cloro/lejía directamente en la pared

Cloro/lejía regada

Cloro/lejía uso

Conocimiento, percepciones y prácticas sobre el uso

de cloro/lejía para prevenir el zika

Conocimiento y percepciones sobre el chikungunya Conocimiento y percepciones sobre el dengue Conocimiento y percepciones sobre el zika Discusión sobre relación entre factibilidad y efectividad de cualquier lámina

Percepción sobre la efectividad del uso de larvicida

(abate, peces, ZAP) en la prevención del zika, según agrupación de láminas

Discusión sobre contraste entre efectividad y

factibilidad del uso de larvicida (abate, peces, ZAP) según agrupamiento de láminas

Percepción sobre la factibilidad del uso de larvicida

(abate, peces, ZAP) en la prevención del zika, según agrupación de láminas

Frecuencia de uso de larvicida según distribución de

abate o ZAP

Conocimiento, percepciones y prácticas sobre el uso de larvicidas (abate, peces, ZAP) para evitar

transmisión de infecciones por vectores Conocimiento, actitudes y prácticas sobre las llantas

en desuso y su relación con el zika

Percepción sobre la efectividad de la eliminación de llantas en desuso en la prevención del zika, según agrupación de láminas

Discusión sobre contraste entre efectividad y

factibilidad de la eliminación de llantas en desuso, según agrupamiento de láminas

Percepción sobre la factibilidad de la eliminación de

llantas en desuso en la prevención del zika, según agrupación de láminas

Mención de otras enfermedades que sufre la

población local, en relación o no al zika

Conocimiento Chikungunya

Conocimiento del Dengue Conocimiento del Zika

Cruce entre factibilidad y efectividad

P. Usar larvicida

Echar larvicida efectividad

Echar larvicida efectividad/factibilidad

Echar larvicida factibilidad

Echar larvicida frecuencia

Echar larvicida uso

J. Eliminar llantas

Eliminación llantas uso

Eliminar llantas efectividad

Eliminar llantas efectividad/factibilidad

Eliminar llantas factibilidad

Enfermedades varias

Q. Fumigar

Fumigación efectividad

Percepción sobre la efectividad de la fumigación en la

prevención del zika, según agrupación de láminas Percepción sobre la factibilidad de la fumigación en la prevención del zika, según agrupación de láminas

Frecuencia y responsable de fumigación en las

viviendas y la comunidad

Conocimiento, percepciones y prácticas sobre la fumigación como medio para evitar transmisión de infecciones por vectores

Percepción sobre la efectividad de la limpieza de

barriles o recipientes grandes de agua en la

prevención del zika, según agrupación de láminas Percepción sobre la efectividad de la limpieza de

barriles o recipientes grandes de agua en la

prevención del zika, según agrupación de láminas Discusión sobre contraste entre efectividad y

factibilidad de la limpieza de barriles o recipientes de agua, según agrupamiento de láminas

Frecuencia y responsable en la familia de la limpieza

de barriles o recipientes de agua

Percepción sobre la efectividad de la limpieza de barriles o recipientes grandes de agua en la

prevención del zika, según agrupación de láminas Discusión sobre contraste entre efectividad y

factibilidad de la limpieza de la pila, según agrupamiento de láminas

Frecuencia, materiales, método y responsable de

limpieza de pila en momentos de epidemia de

enfermedades transmitidas por vectores Frecuencia, materiales, método y responsable de

limpieza de pila según la estación: seca o lluviosa Percepción sobre la factibilidad de la limpieza de barriles o recipientes grandes de agua en la

prevención del zika, según agrupación de láminas

Fumigación factibilidad

Fumigación frecuencia

Fumigación razón

G. Limpiar recipientes grandes de agua (barriles)

Limpieza de barriles efectividad

Limpieza de barriles factibilidad

limpieza recipientes

efectividad/factibilidad

Limpieza de barriles frecuencia

O. Limpiar la pila

Limpieza de pila efectividad

Limpieza de pila efectividad/factibilidad

Limpieza de pila en momentos de

epidemia

Limpieza de pila estacionalidad

limpieza de pila factibilidad

Limpieza de pila frecuencia

Frecuencia y responsable en la familia de la limpieza

de la pila

Momentos y formas en que otras personas, que no son la mujer responsable del hogar, ayudan a limpiar la pila o sugerencias al respecto

Responsable, frecuencia, materiales y método de

limpieza de pila cuando la mujer responsable de la limpieza de la pila está embarazada

Frecuencia, materiales y método de limpieza de pila

empleado por los hombres

Frecuencia, materiales y método de limpieza de pila empleado por las mujeres

Materiales, métodos y frecuencia empleados en la

limpieza de la pila según el tamaño de la misma Percepción sobre la efectividad de la limpieza de áreas comunes en la comunidad en la prevención del zika,

según agrupación de láminas

Percepción sobre la factibilidad de la limpieza colectiva

de áreas comunes en la comunidad en la prevención

del zika, según agrupación de láminas Conocimiento, percepciones y prácticas sobre la

limpieza colectiva de la comunidad como medio para

evitar transmisión de infecciones por vectores Percepción sobre la efectividad del uso de manga

larga entre mujeres embarazadas para prevenir el zika, según agrupación de láminas

Percepción sobre la factibilidad del uso de manga

larga entre mujeres embarazadas para prevenir el zika, según agrupación de láminas

Conocimiento, actitudes y prácticas sobre el uso de

manga larga por embarazadas como medio para evitar transmisión de infecciones por vectores

Limpieza de pila motivar a otros

Limpieza de pila por embarazadas

Limpieza de pila por hombre

Limpieza de pila por mujeres

limpieza pila adaptación tamaño pila

K. Limpieza en comunidad

Limpieza en comunidad efectividad

Limpieza en comunidad factibilidad

Limpieza en comunidad razones

M. Usar manga larga

Manga larga efectividad

Manga larga factibilidad

Manga larga uso

H. Usar zancudero/mosquitero

Mosquitero/Zancudero efectividad

Percepción sobre la efectividad del uso de

mosquiteros o zancuderos para prevenir el zika, según agrupación de láminas

Discusión sobre contraste entre efectividad y

factibilidad del uso de mosquitero o zancudero para

prevenir el zika, según agrupamiento de láminas Percepción sobre la factibilidad del uso de

mosquiteros o zancuderos para prevenir el zika, según agrupación de láminas

Conocimiento, actitudes y prácticas sobre el uso del

mosquitero o zancudero como medio para evitar

transmisión de infecciones por vectores Percepción sobre la efectividad de la abstención

sexual para prevenir el zika, según agrupación de láminas

Discusión sobre contraste entre efectividad y

factibilidad de la abstención sexual para prevenir el zika, según agrupamiento de láminas

Percepción sobre la factibilidad de la abstención

sexual para prevenir el zika, según agrupación de láminas

Conocimiento y relación de la población con

proyectos de control vectorial realizados por ongs nacionales o internacionales

Conocimiento y relación de la población con

proyectos de control vectorial realizados por instituciones gubernamentales de carácter municipal o nacional

Preocupación de las familias sobre el contagio del zika

al momento de las entrevistas o grupos focales Preocupación de las familias sobre el contagio del zika cuando comenzaron a oir sobre la enfermedad

Mosquitero/zancudero

efectividad/factibilidad

Mosquitero/Zancudero factibilidad

Mosquitero/zancudero uso

L. No tener relaciones sexuales

No tener relaciones sexuales efectividad

No tener relaciones sexuales

efectividad/factibilidad

No tener relaciones sexuales factibilidad

Control de vectores

Papel de ONG en control vectorial

Papel del Estado en control vectorial

Preocupación actual sobre Zika

Preocupación inicial sobre Zika

F. Usar preservativo durante el embarazo

Preservativo durante el embarazo

efectividad

Percepción sobre la efectividad del uso del condón o

preservativo para prevenir el zika, según agrupación de láminas

Percepción sobre la factibilidad del uso del condón o

preservativo para prevenir el zika, según agrupación de láminas

Discusión sobre contraste entre efectividad y

factibilidad del uso del preservativo o condón durante

el embarazo, según agrupamiento de láminas Percepciones y actitudes de la mujer sobre la toma de

decisión de uso del preservativo

Percepción sobre la efectividad del uso de raquetas que electrocutan mosquitos para la prevención del zika

Lógica de agrupación de todas las láminas clasificadas

como más o menos efectivas

Lógica de agrupación de todas las láminas clasificadas como muy efectivas

Lógica de agrupación de todas las láminas clasificadas

como poco efectivas

Lógica de agrupación de todas las láminas clasificadas como más o menos factibles

Lógica de agrupación de todas las láminas clasificadas

como muy factibles

Lógica de agrupación de todas las láminas clasificadas como poco factibles

Percepción sobre la efectividad de recoger la basura

(limpieza seca) frente a la vivienda para prevenir el zika, según agrupación de láminas

Discusión sobre contraste entre efectividad y

factibilidad de recoger basura (limpieza seca) frente a la vivienda, según agrupamiento de láminas

Preservativo durante el embarazo

factibilidad

Preservativo efectividad/factibilidad

Preservativo negociación pareja

percepción de mujer Raquetas efectividad

Razones de agrupamiento por

efectividad más o menos Razones de agrupamiento por efectividad mucho

Razones de agrupamiento por

efectividad poco

Razones de agrupamiento por factibilidad más o menos Razones de agrupamiento por

factibilidad mucho

Razones de agrupamiento por factibilidad poco

Recoger basura efectividad

R. Recoger basura alrededor de la casa

Recoger basura efectividad/factibilidad

Recoger basura factibilidad

Percepción sobre la factibilidad de recoger la basura

(limpieza seca) frente a la vivienda para prevenir el zika, según agrupación de láminas

Motivos, frecuencia y responsable de recoger la

basura frente a la vivienda como un medio para evitar

la transmisión de enfermedades por vectores Percepción sobre la efectividad de utilizar repelente

para prevenir el zika, según agrupación de láminas Discusión sobre contraste entre efectividad y factibilidad de utilizar repelente para prevenir el zika,

según agrupamiento de láminas

Percepción sobre la factibilidad de utilizar repelente

para prevenir el zika, según agrupación de láminas Conocimientos, percepciones y prácticas sobre el uso de repelente de uso personal o de otro tipo

(sahumerios, laminillas, aután)

Comentarios sobre el uso de materiales de limpieza

según el tamaño de la pila durante el simulacro de limpieza

Comentarios sobre método de limpieza según el

tamaño de la pila durante el simulacro de limpieza Comentarios sobre la aplicación de cloro/lejía en el agua de la pila o mezclado con agua durante el

simulacro de limpieza

Comentarios sobre la aplicación de larvicida en el agua

de la pila durante el simulacro de limpieza Comentarios sobre la aplicación de cloro/lejía directamente a la pila durante el simulacro de

limpieza

Comentarios sobre untar lejía a la pila durante el

simulacro de limpieza

Comentarios sobre la cantidad de agua utilizada para limpiar la pila durante el simulacro de limpieza

recoger basura razón

A. Usar repelente

Repelente efectividad

Repelente efectividad/factibilidad

Repelente en la piel factibilidad

Repelente uso

Simulacro adaptación de materiales

según tamaño de pila

Simulacro adaptación de proceso según

tamaño de pila

Simulacro aplicación de lejía en el agua

Simulacro aplicación larvicida

Simulacro aplicación lejía sin agua

Simulacro aplicación lejía untada

Simulacro cantidad de agua

Simulacro cantidad de lejía

Comentarios sobre la cantidad de cloro/lejía utilizada

para limpiar la pila durante el simulacro de limpieza Comentarios sobre la cantidad de detergente utilizada para limpiar la pila durante el simulacro de limpieza

Simulacro cantidad detergente

Simulacro limpieza de pila afuera

Comentarios sobre la limpieza por la parte de afuera

de la pila durante el simulacro de limpieza Comentarios sobre los materiales utilizados para la limpieza de la pila durante el simulacro de limpieza

Comentarios sobre el método de restregado para la

limpieza de la pila durante el simulacro de limpieza Comentarios sobre el momento para tapar el desagüe de la pila mientras se limpia durante el simulacro de

limpieza

Comentarios sobre el tiempo que se deja reposando el

cloro para la limpieza de la pila durante el simulacro de limpieza

Comentarios sobre el momento, forma y tiempo de la

untadita para limpieza de la pila, durante el simulacro de limpieza

Percepción sobre la efectividad de tapar los

recipientes donde se guarda agua para prevenir el zika, según agrupación de láminas

Percepción sobre la factibilidad de tapar los

recipientes donde se guarda agua para prevenir el zika, según agrupación de láminas

Discusión sobre contraste entre efectividad y

factibilidad de tapar los recipientes donde se guarda agua para prevenir el zika, según agrupamiento de láminas

Descripción de tipo y tamaños de pila utilizados

(incluye otros tipos que no son propiamente pilas pero son usados como tal)

Simulacro limpieza pila materiales

Simulacro restregado de pila

Simulacro tapar pila

Simulacro tiempo reposo lejía

Simulacro untadita

D. Tapar recipientes de almacenamiento de agua

Tapar recipientes de almacenamiento

de agua efectividad

Tapar recipientes de almacenamiento

de agua factibilidad

Tapar recipientes

efectividad/factibilidad

Tipos de pila

N. Untar lejía a los barriles

Untar lejía a los barriles efectividad

Percepción sobre la efectividad de untar cloro/lejía a

los barriles lo recipientes grandes de agua para

prevenir el zika, según agrupación de láminas Percepción sobre la factibilidad de untar cloro/lejía a

los barriles o recipientes grandes de agua para

prevenir el zika, según agrupación de láminas Discusión sobre contraste entre efectividad y

factibilidad de untar cloro/lejía en barriles o recipientes grandes para guardar agua para prevenir el zika, según agrupamiento de láminas

Percepción sobre la efectividad de dar vuelta o vaciar

el agua de recipientes en el patio para prevenir el zika, según agrupación de láminas

Motivos, frecuencia y responsable de dar vuelta o

vaciar el agua de recipientes en el patio como un medio para evitar la transmisión de enfermedades por vectores

Discusión sobre contraste entre efectividad y

factibilidad dar vuelta o vaciar el agua de recipientes en el patio para prevenir el zika, según agrupamiento de láminas

Percepción sobre la factibilidad de dar vuelta o vaciar

el agua de recipientes en el patio para prevenir el zika, según agrupación de láminas

Conocimientos y percepciones sobre el nacimiento y

reproducción de los mosquitos o zancudos (sin distinguir especie)

Conocimientos y percepciones sobre la relación de los

mosquitos o zancudo y el agua (sin distinguir especie)

Conocimientos y percepciones sobre los tipos de enfermedades transmitidas por los zancudos o mosquitos

Untar lejía a los barriles factibilidad

Untar lejía efectividad/factibilidad

B. Vaciar recipentes no intencionales del patio

Vaciar recipientes del patio efectividad

Vaciar recipientes del patio uso

Vaciar recipientes

efectividad/factibilidad

Vaciar recipientes factibilidad

Zancudo cualquiera nacimiento

Zancudo cualquiera tipo de agua

asociado

Zancudo enfermedades

I. Usar zaranda en ventanas y puertas

Zaranda/malla efectividad/factibilidad

Discusión sobre contraste entre efectividad y

factibilidad de usar zaranda o malla en las puertas y ventanas de la vivienda para prevenir el zika, según agrupamiento de láminas

Percepción sobre la efectividad de usar zaranda o

malla en puertas y ventanas de la vivienda para

prevenir el zika, según agrupación de láminas Percepción sobre la factibilidad de usar zaranda o

malla en puertas y ventanas de la vivienda para prevenir el zika, según agrupación de láminas

Zaranda/malla efectividad

Zaranda/malla factibilidad
