# Supplementary material for: A qualitative study of community perspectives surrounding cleaning practices in the context of Zika prevention in El Salvador: implications for community-based Aedes aegypti control
Source: BMC Public Health. 2020 Sep 11;20:1385. doi: 10.1186/s12889-020-09370-5 (PMC7488301; doi:10.1186/s12889-020-09370-5)
Supplement: Supplementary file 6 — Additional file 6. PUBH-D-20-01205 Codebook May 2018 English translation.docx [file 12889_2020_9370_MOESM6_ESM.docx]

Use of bleach

**Code**   **groups**

**Codes**

Water supply

**Code explanation**

Frequency and amount of water to which families have access, either through buying or collecting rainwater or river

Perception of the effectiveness of the methods of

family planning in Zika prevention, according to clustering of the cards

Discussion on contrast between effectiveness and

feasibility of family planning methods according to clustering of cards

Feasibility perception of methods of

family planning in Zika prevention, according to clustering of cards

Using family planning methods

Perception of prenatal control effectiveness for Zika prevention

Discussion on contrast between effectiveness and

feasibility of prenatal control according to clustering of cards

Feasibility perception of prenatal control in

Zika prevention, according to clustering of cards knowledge, attitudes and practices regarding prenatal control

Chlorine/bleach application directly on the wall of

the water container (untadita and other forms of application)

Application of chlorine/bleach directly from the bag to the walls

walls of the pile or recipiiwater

E. Family planning methods

Contraceptives/ Family Planning

Effectiveness

Contraceptives/ Family Planning

effectiveness/feasibility

Contraceptives/ Family Planning

Feasibility

Contraceptives/Family Planning Use

Attending prenatal care effectiveness

C. Attending prenatal care

Attend prenatal care

effectiveness/feasibility

Attending prenatal care feasibility

Attend prenatal care use

Chlorine/bleach directly on the wall

Chlorine poured

Eliminate tires effectiveness//feasibility

Eliminate tires effectiveness

To put larvicide - effectiveness effectiveness

larvicide feasibility and effectiveness

Chikungunya Knowledge

Knowledge of The Knowledge of Zika

A cross between feasibility and effectiveness

Chlorine use

Knowledge, perceptions and practices about use of

chlorine/bleach to prevent Zika

Knowledge and perceptions about chikungunya Knowledge and perceptions about dengue Knowledge and perceptions about Zika

Discussion on the relationship between feasibility and effectiveness of any cards

Perception of the effectiveness of larvicide use

(abate, fish, ZAP) in Zika prevention, according to clustering of cards

Discussion on contrast between effectiveness and

feasibility of the use of larvicide (abate, fish, ZAP) according to grouping of sheets

Perception of the feasibility of larvicide use

(abate, fish, ZAP) in Zika prevention, according to clustering of cards

Frequency of use of larvicide according to distribution of abate or Zap

Knowledge, perceptions and practices on the use of larvicides (abate, fish, ZAP) to avoid

vector infections

Knowledge, attitudes and practices on unused tires

and their relationship with Zika

Perception of the effectiveness of unused tire removal for Zika prevention, according to card groupings

Discussion on contrast between effectiveness and

feasibility of removing disused tires, according to card clustering

Perception of the feasibility of eliminating

disused tires for Zika prevention, according to clustering of cards

Mention of other diseases suffered by the

local population, whether or not related to Zika

Q. Use larvicide

To pour larvicide - feasibility effectiveness/feasibility

larvicide frequency

larvicide use

J. Remove tires

Removal tires use

Eliminate feasibility tires

Various diseases

cleaning containers

effectiveness/feasibility

O. Clean the pila

Fumigation reasons

Fumigation effectiveness

Q. Fumigate

Perception of the effectiveness of fumigation in

Zika prevention, according to grouping of sheets Perception on the feasibility of fumigation in Zika prevention, according to clustering of cards

Frequency and responsible for fumigation in

housing and the community

Knowledge, perceptions and practices about fumigation as a means to prevent vector infection transmission

Perception about the effectiveness of cleaning

barrels or large containers of water for

Zika prevention, according to grouping of cards Perception on the effectiveness of cleaning

barrels or large containers of water in the

Zika prevention, according to card grouping Discussion on contrast between effectiveness and

feasibility of cleaning barrels or water containers, depending on the grouping of cards

Frequency and responsible in the cleaning family

barrels or containers of water

Perception about the effectiveness of cleaning large barrels or containers of water in the

Zika prevention, according to film grouping Discussion on contrast between effectiveness and

feasibility of cleaning the pila, according to clustering of cards

Frequency, materials, methods and responsibles for

pila cleaning at times of epidemics

Frequency, materials, methods and responsibles of pila cleaning in dry or rainy season

Perception about the feasibility of cleaning barrels or large containers of water for

Zika prevention, according to clustering of cards

Fumigation feasibility

Fumigation frequency

G. Clean large containers of water (barrels)

Cleaning barrels effectively

Cleaning barrels feasibility

Cleaning barrels frequency

Pila cleaning effectiveness

Pila cleaning effectiveness/feasibility

Pila cleaning at times of

Epidemic

Seasonal pila cleaning

Pila cleaning feasibility leaning

Long-sleeve effectiveness

Cleaning the pila by pregnant women

Frequency of pila cleaning

Frequency and responsibles in the cleaning of pila in the family

Moments and ways in which other people, who are not the woman responsible at the home, help clean the pila or suggestions about it

Responsible, frequency, materials and methods of

cleaning pila when the woman responsible for cleaning the pila is pregnant

Frequency, materials and stack cleaning method

employed by men

Frequency, materials and methods of cleaning pila used by women

Materials, methods and frequency used in the

cleaning the pila according to the size of the same Perception on the effectiveness of cleaning common areas in the community in Zika prevention,

according to grouping of cards

Perception of the feasibility of collective cleaning

common areas in the community for prevention of

Zika, according to cluster of cards

Knowledge, perceptions and practices on Zika

collective cleanliness of the community as a means to

prevent transmission of vector infections Perception about the effectiveness of long sleeve use by pregnant women to prevent Zika, according to clustering of cards

Perception of the feasibility of long sleeve use by

pregnant women to prevent Zika, according to clustering of cards

Knowledge, attitudes and practices about the use of

long sleeves by pregnant women as a means to prevent vector infection transmission

Cleaning pila motivate others

pila cleaning by men

Pila cleaning by women

cleaning pila according to size

K. Community Cleanup

Community cleanup effectiveness

Community cleanup feasibility

Community cleanup reasons

M. Wear long sleeves

Long sleeve feasibility sleeve

Long sleeve use

Initial concern about Zika

Current concern about Zika

Mosquito net use

Mosquito net

effectiveness/feasibility

H. Use of mosquito net

Mosquito net effectiveness

Perception of the effectiveness of the use of

mosquito nets to prevent Zika, according to clustering of cards

Discussion on contrast between effectiveness and

feasibility of using a mosquito net to prevent Zika, according to clustering of cards

Perception about the feasibility of using Zika

mosquito nets or mosquito nets to prevent Zika, according to clustering of cards

Knowledge, attitudes and practices on the use of

mosquito net as a means to avoid transmission of vector infections

Perception of the effectiveness of abstinence to prevent Zika, according to clustering of cards

Discussion on contrast between effectiveness and

feasibility of sexual abstinence to prevent Zika, according to clustering of cards

Perception of the feasibility of abstinence to prevent Zika, according to clustering of cards

Knowledge and relationship of the population with

vector control projects carried out by national or international ngos

Knowledge and relationship of the population with

vector control projects carried out by municipal or national government institutions

Families' concern about Zika infection at the time of interviews or focus groups

Families' concern about Zika infection when they began to hear about the disease

Mosquito net feasibility

L. Not having sex

Not having sex - effectiveness

Not having sex

effectiveness/feasibility

Not having sex feasibility

Vector control

Role of NGOs in vector control

State's role in vector control

Picking up trash effectiveness/feasibility

F. Use condoms during pregnancy

Condom during pregnancy

Effectiveness

Perception about the effectiveness of condom use or

condom to prevent Zika, according to clustering of cards

Perception of the feasibility of condom use or

condom to prevent Zika, according to clustering of cards

Discussion on contrast between effectiveness and

feasibility of condom or condom use during

pregnancy, according to clustering of pictures Perceptions and attitudes of women about taking the

decision to use the condom

Grouping logic of all cards classified as more or less effective

Grouping logic of all cards classified as very effective

Grouping logic of all classified cards as ineffective

Grouping logic of all cards classified as more or less feasible

Grouping logic of all classified cards as very feasible

Grouping logic of all cards classified as impractical

Perception about the effectiveness of picking up trash

in front of the dwelling to prevent Zika, according to clustering of cards

Discussion on contrast between effectiveness and

feasibility of collecting trash in front of the house, according to grouping of cards

Condom during pregnancy

Feasibility

Condom effectiveness/factibillity

Condom negotiation with partner perception of women effectiveness

Reasons for grouping by

effectiveness more or less Reasons for grouping as very effective

Reasons for grouping as ineffective

Reasons for grouping by feasibility as more or less Reasons for grouping by

feasibility as a lot

Reasons for grouping for poor feasibility

Picking up trash effectiveness

A. Picking up trash around the house

Simulation amount of water

Simulation process adaptation according to pila size

Simulation application of bleach in the water

Simulation materials according to pila size

Repellent use

Repellent effectiveness/feasibility

Repellent effectiveness

pick up garbage reasons

Picking up trash feasibility garbage

Perception of the feasibility of picking up trash

in front of the dwelling to prevent Zika, according to clustering of cards

Reasons, frequency and responsible for collecting the

trash in front of housing as a means to avoid

vector disease transmission

Perception about the effectiveness of using repellent to prevent Zika, according to card groupings

Discussion on contrast between effectiveness and feasibility of using repellent to prevent Zika,

according to grouping of cards

Perception of the feasibility of using repellent

to prevent Zika, according to card groupings Knowledge, perceptions and practices on the use of repellent for personal or other use

Comments on the use of cleaning materials

depending on the size of the pila during the simulation

Comments on cleaning method according to pila size during the simulation

Reviews on the application of chlorine/bleach in the water of the pila or mixed with water during the

simulation of cleaning

Comments on the application of larvicide in water

during the simulation of cleaning

Comments on the application of chlorine/ bleach directly to the pila during the simulation of cleaning

Comments on spreading bleach to the pila walls during the simulation of cleaning

Comments on the amount of water used to clean the pila during the cleaning simulation

A. Use repellent

Skin repellent feasibility

Simulation larvicide application

Simulation application bleach without water

Simulation app bleach smeared

Pila types

Covering containers

effectiveness/feasibilityfactibilidad

Simulation - time resting of bleach

Simulation of covering the pila drain

Simulation pila cleaning materials

Simulated pila cleaning on the outside

Simulation amount of detergent

Simulation amount of bleach

Comments on the amount of chlorine/bleach used

to clean the pila during the cleaning simulation Comments on the amount of detergent used to clean the pila during the cleaning simulation

Comments on cleaning on the outside of the pila during the simulation cleaning

Comments on the materials used for cleaning the pila during the cleaning simulation

Comments on the scrubbing method for

cleaning the pila during the simulation cleaning Comments on plugging of the drain of the pila while cleaning during the simulation cleaning

Comments on the time bleach was left resting when cleaning the pila during the cleaning simulation

Comments on the timing, form and time of the

untadita for cleaning the pila, during the cleaning simulation

Perception of the effectiveness of covering

water storage containers to prevent Zika, according to clustering of cards

Perception of the feasibility of covering

water storage containers to prevent Zika, according to clustering of cards

Discussion on contrast between effectiveness and

feasibility of covering water storage containers to prevent Zika, according to clustering of cards

Description of type and sizes of pilas used

(includes other types that are not proper pilas but are used as such)

Pila scrubbing simulation

Untadita simulation

D. Covering water storage containers

Covering storage containers

water effectiveness

Covering storage containers

water feasibility

Mosquito borne diseases

Mosquitoes associated with any type of water

Birth of any type of mosquitoes

Emptying containers feasibility

Empty containers

effectiveness/feasibility

Emptying out yard containers use

Emptying out yard containers effectiveness

Spread bleach effectiveness//feasibility

Spreading bleach to barrels effectiveness

N. Spread bleach to barrels

Perception about the effectiveness of spreading chlorine/bleach to barrels and large containers of water to prevent Zika, according to grouping of cards Perception on the feasibility of spreading chlorine/bleach to barrels or large containers of water to prevent Zika, according to cluster of cards

Discussion on contrast between effectiveness and

feasibility of spreading chlorine/bleach in large barrels or recipients to store water to prevent Zika, according to clustering of cards

Perception of the effectiveness of turning or emptying

water from containers in the yard to prevent Zika, as grouped with cards

Reasons, frequency and responsible for turning or

emptying water from containers in the yard as a means to prevent vector disease transmission

Discussion on contrast between effectiveness and

feasibility to turn or empty water from containers in the yard to prevent Zika, according to clustering of cards

Perception of the feasibility of turning or emptying

water from containers in the yard to prevent Zika, as grouped with cards

Knowledge and perceptions about birth and

reproduction of mosquitoes (without distinguishing species)

Knowledge and perceptions about the relationship of

mosquitoes with water (without distinguishing species)

Knowledge and perceptions of the types of diseases transmitted by mosquitoes

Spread bleach to barrels feasibility

B. Empty water from unintentional containers in the yard

I. Use screens on windows and doors

screen/mesh effectiveness/feasibility

Discussion on contrast between effectiveness and

feasibility of using screen or mesh on the doors and windows of the house to prevent Zika, according to clustering of cards

Perception about the effectiveness of using screen or mesh in doors and windows of the house to

prevent Zika, according to grouping of cards Perception about the feasibility of using screen or

mesh on doors and windows of the house to prevent Zika, according to clustering of cards

screen/mesh effectiveness

screen/mesh feasibility
