# Supplementary material for: A qualitative study of community perspectives surrounding cleaning practices in the context of Zika prevention in El Salvador: implications for community-based Aedes aegypti control
Source: BMC Public Health. 2020 Sep 11;20:1385. doi: 10.1186/s12889-020-09370-5 (PMC7488301; doi:10.1186/s12889-020-09370-5)
Supplement: Supplementary file 7 — Additional file 7. PUBH-D-20-01205 COREQ list.docx [file 12889_2020_9370_MOESM7_ESM.docx]

**PUBH-D-20-01205 Supplementary Information**

Leontsini et al. A Qualitative Study of Community Perspectives Surrounding Cleaning Practices in the Context of Zika Prevention in El Salvador: Implications for Community-Based *Aedes aegypti* Control

**Consolidated criteria for reporting qualitative studies (COREQ): 32-item checklist^[[1]](#endnote-1)^**

| **No .** | **Item .** | **Guide questions/**  **description .** | **Response** |
| --- | --- | --- | --- |
| **Domain 1: Research team and reflexivity** | | | |
| Personal Characteristics | | | |
| 1. | Interviewer/  facilitator | Which author/s conducted the interview or focus group? | Ramírez M, Leontsini E, Maloney S |
| 2. | Credentials | What were the researcher's credentials? *E.g. PhD, MD* | Leontsini E, MD, MPH in International Health  Maloney S, MPH in Social and Behavioral Sciences and Health Communication  Ramírez M, PhD in Political Science and Sociology  Rodriguez E, MSPH student in Social and Behavioral Interventions in International Health  Gurman T, DrPH in Health Communication in Population and Family Health Sciences, MPH in Maternal and Child Health  Ballard Sara A, MPH in Global Health and Communication  Hunter GC, MHS in Social and Behavioral Interventions in International Health |
| 3. | Occupation | What was their occupation at the time of the study? | Leontsini E: Associate Scientist at the Social and Behavioral Interventions Program, Dept of International Heath, Johns Hopkins Bloomberg School of Public Health  Maloney S: Program Officer II, Johns Hopkins Center for Communication Programs, Bloomberg School of Public Health  Ramírez M: Independent Consultant, Guatemala City, Guatemala  Rodriguez E: Research Assistant, Johns Hopkins Center for Communication Programs, Bloomberg School of Public Health  Gurman T: Research and Evaluation Officer, Johns Hopkins Center for Communication Programs, Bloomberg School of Public Health  Ballard Sara A: Program Officer II, Johns Hopkins Center for Communication Programs, Bloomberg School of Public Health  Hunter GC: Senior Technical Advisor, Johns Hopkins Center for Communication Programs, Bloomberg School of Public Health |
| 4. | Gender | Was the researcher male or female? | Two researchers self-identify as male and four researchers as female |
| 5. | Experience  and training | What experience or training did the researcher have? | Leontsini, E: over 25 years of experience teaching qualitative and formative research methods at JHSPH, leading qualitative and formative research, and collecting and analyzing qualitative data in public health studies  Maloney, S: 5 years of experience collecting qualitative data in public health research, as a Safe Water and Medical Program Coordinator and as Program Officer; 3 years of experience training field researchers on qualitative data collection  Ramírez, M: Over 20 years of experience supervising qualitative studies, training field researchers and collecting and analyzing qualitative data in public health studies  Rodriguez E: 1 year of training in qualitative and formative research methods in public health, literature reviews and scientific writing  Ballard Sara A: 8 years of experience collecting qualitative data in public health research and training field researchers on qualitative data collection  Gurman T: Over 15 years of experience leading qualitative research, training data collectors, as well as collecting and analyzing qualitative data  Hunter GC: 15 years of experience supervising qualitative studies, training field researchers and collecting and analyzing qualitative data in public health studies  All members of the research team were native or bilingual Spanish speakers and familiar with the Central American context. |
| Relationship with participants | | | |
| 6. | Relationship established | Was a relationship established prior to study commencement? | USAID’s Zika implementing partners with whom we collaborated in participant recruitment had established a relationship with a majority of participants through behavior change community education and mobilizing activities; the rest were recruited at health centers by health center staff on behalf of USAID’s Zika implementing partners and did not necessarily have an established relationship with these partners. |
| 7. | Participant knowledge of  the interviewer | What did the participants know about the researcher? e*.g. personal goals, reasons for doing the research* | Through our oral recruitment script and oral consent form, participants knew that we were from Johns Hopkins University in the United States, conducting a study on the measures of Zika prevention in the community, with the goal to contribute to the improvement of future Zika prevention programs. After doing introductions at the beginning of each focus group or IDI the participants knew us in person, and in addition that we had come from the US or from Guatemala specifically for the study. |
| 8. | Interviewer characteristics | What characteristics were reported about the interviewer/facilitator? e.g. *Bias, assumptions, reasons and interests in the research topic* | We reported to the participants that we were social scientists, very interested in what they had to say about Zika, with the goal of contributing to improved Zika prevention programs in the future. We were vigilant to ask non-leading questions and put our biases or assumptions aside if we had any. We created a positive and welcoming atmosphere. We encouraged participants to freely express their ideas and perceptions about their communities, with regard to Zika and its prevention. |
| **Domain 2: study design** | | | |
| Theoretical framework | | | |
| 9. | Methodological orientation and Theory | What methodological orientation was stated to underpin the study? *e.g. grounded theory, discourse analysis, ethnography, phenomenology, content analysis* | Our theoretical framework came from behavioral psychology on factors influencing behavior and behavior change and we cited Fishbein et al (ref 29). We studied men and women separately thus exploring gender. We studied pregnant women separately from non-pregnant women to further explore risk perception and behavior change. Our methodology had both inductive elements (free elicitation of community practices) and deductive exploration of predefined themes (effectiveness, feasibility, intentions) while embracing any other emerging themes like e.g. seasonality. |
| Participant selection | | | |
| 10. | Sampling | How were participants selected? *e.g. purposive, convenience, consecutive, snowball* | Participants were selected by purposive convenience sampling |
| 11. | Method of approach | How were participants approached? e*.g. face-to-face, telephone, mail, email* | Participants were approached face-to-face by USAID´s Zika implementing partner, health centers staff and the researchers |
| 12. | Sample size | How many participants were in the study? | 11 Focus groups and 12 interviews  Focus group participants: 32 pregnant women, 24 non-pregnant women, 15 men  Interviews: 12 men  Urban area: 40 participants  Rural area: 43 participants  Total: 83 participants |
| 13. | Non-participation | How many people refused to participate or dropped out? Reasons? | Convenience sampling was drawn by USAID implementing partners who did not keep records of refusals. Focus groups were held in a facility near the health center. Pregnant women participated before or after their antenatal care visit. A few of them left before the focus group ended in order to make their appointment. A few other participants left early for a variety of other commitments. A total of 6 participants left early. |
| Setting | | | |
| 14. | Setting of data collection | Where was the data collected? e*.g. home, clinic, workplace* | Data was collected at Municipality or health center facilities in independent meeting rooms separated from clinics or waiting areas. |
| 15. | Presence of non-participants | Was anyone else present besides the participants and researchers? | No. In one focus group, men waited for their participating pregnant wives at a considerable distance. |
| 16. | Description of sample | What are the important characteristics of the sample? *e.g. demographic data, date* | There were three groups of participants: pregnant women, male partners of pregnant women, and young non-pregnant women likely to become pregnant. Cases in which both the pregnant woman and her male partner participated in the study, were rare exceptions.  The study took place at two localities: one urban, in a small town 37 miles from the capital city; the other rural and very small town at 60 miles from the capital city.  The characteristics of the participants are illustrated in Table 1. |
| Data collection | | | |
| 17. | Interview guide | Were questions, prompts, guides provided by the authors? Was it pilot tested? | Yes, with inputs from other participating staff, mentioned in the acknowledgments. Experience had been drawn from a previous study conducted in Guatemala by four of the same authors. The guides were therefore pilot tested in house only, and a few adjustments were made due to differences in vocabulary. |
| 18. | Repeat interviews | Were repeat interviews carried out? If yes, how many? | No repeat interviews were carried out. |
| 19. | Audio/visual recording | Did the research use audio or visual recording to collect the data? | The research used audio recording to capture discussions and dialogues, as well as visual recording to capture cleaning simulations. |
| 20. | Field notes | Were field notes made during and/or after the interview or focus group? | Field notes were made both during and after the interviews and focus groups. |
| 21. | Duration | What was the duration of the interviews or focus group? | The duration of the interviews and focus groups was one hour to one hour and a half each. |
| 22. | Data saturation | Was data saturation discussed? | Data saturation was discussed in the Discussion section, first paragraph. |
| 23. | Transcripts returned | Were transcripts returned to participants for comment and/or correction? | Transcripts were not returned to participants – we did not collect participants’ personal identifiers and further follow up was impossible by design. |
| **Domain 3: analysis and findings** | | | |
| Data analysis | | | |
| 24. | Number of data coders | How many data coders coded the data? | 2 coders |
| 25. | Description of the coding tree | Did authors provide a description of the coding tree? | Our coding tree is provided as supplemental material. |
| 26. | Derivation of themes | Were themes identified in advance or derived from the data? | Themes were identified mainly in advance and secondarily derived from the data. |
| 27. | Software | What software, if applicable, was used to manage the data? | Atlas.ti (textual data) and Excel (ratings and votes) |
| 28. | Participant checking | Did participants provide feedback on the findings? | Participants did not provide feedback on the findings– we did not collect participants’ personal identifiers and further follow up was impossible by design. An analysis workshop took place with USAID´s Zika implementers that provided further insight into Zika situation in El Salvador and prevention measures promoted by the implementing partners. A presentation of initial findings to Zika implementing partners and Ministry of Health staff generated a discussion on how to improve Zika prevention communication campaigns in the future. |
| Reporting | | | |
| 29. | Quotations presented | Were participant quotations presented to illustrate the themes / findings? Was each quotation identified? e*.g. participant number* | We have presented several participant quotations to illustrate the themes. Each quotation was identified. |
| 30. | Data and findings consistent | Was there consistency between the data presented and the findings? | There was high consistency between the data presented and the findings. |
| 31. | Clarity of major themes | Were major themes clearly presented in the findings? | Major themes were clearly presented in the findings. |
| 32. | Clarity of minor themes | Is there a description of diverse cases or discussion of minor themes? | We have included a description of diverse cases and minor themes throughout. |

[Open in new tab](https://academic.oup.com/view-large/%5bXSLTSectionID%5d%5bQueryString%5d)

1. Tong A, Sainsbury P, Craig J. **Consolidated criteria for reporting qualitative research (COREQ): a 32-item checklist for interviews and focus groups**, International Journal for Quality in Health Care, Volume 19, Issue 6, December 2007, Pages 349–357, <https://doi.org/10.1093/intqhc/mzm042> [↑](#endnote-ref-1)
